# Characterization of Cell-Envelope Proteinases from Two *Lacticaseibacillus casei* Strains Isolated from Parmigiano Reggiano Cheese

**DOI:** 10.3390/biology11010139

**Published:** 2022-01-14

**Authors:** Lisa Solieri, Laura Sola, Amanda Vaccalluzzo, Cinzia Lucia Randazzo, Serena Martini, Davide Tagliazucchi

**Affiliations:** 1 Department of Life Sciences, University of Modena and Reggio Emilia, via Amendola, 2—Pad. Besta, 42100 Reggio Emilia, Italy; lisa.solieri@unimore.it (L.S.); laura.sola@unimore.it (L.S.); serena.martini@unimore.it (S.M.); 2 Department of Agriculture, Food and Environment, University of Catania, via Santa Sofia, 100, 95123 Catania, Italy; amanda.vaccalluzzo@unict.it (A.V.); cranda@unict.it (C.L.R.); 3 ProBioEtna srl, Spin off University of Catania, via Santa Sofia, 100, 95123 Catania, Italy

**Keywords:** lactic acid bacteria, fermented food, functional food, bioactive peptides, protease, peptidomics

## Abstract

**Simple Summary:**

Lactic acid bacteria are nutritionally fastidious microorganisms typically used in the production of fermented dairy foods. The lactic acid bacteria’s proteolytic system is crucial for their growth in milk, and plays a paramount role in developing the organoleptic and healthy properties of fermented dairy foods. Cell-envelope proteinases are the first component of this system, responsible for the degradation of caseins into short peptides. In the present work, the cell-envelope proteinases of two highly proteolytic *Lacticaseibacillus casei* strains, previously isolated from ripened Parmigiano Reggiano cheese, were characterized from a genetic and biochemical point of view. Two different *prt* genes existed in the genomes of both strains, but only one, named *prtR1*, was expressed. PrtR1 proteins extracted from both strains displayed the highest activity at 40 °C and pH 7. Interestingly, PrtR1 extracted from *Lacticaseibacillus casei* PRA205 retained some residual activity at 5 °C and at pH 4. These important biotechnological features can be exploited in the production of fermented dairy products. Peptidomic analysis revealed that both proteinases were able to release β- and αS1-casein-derived bioactive peptides, suggesting that *Lacticaseibacillus casei* can be a source of new proteinases that can be exploited for the formulation of dairy beverages or hydrolysates enriched in bioactive peptides.

**Abstract:**

In the present work, two cell-envelope proteinases (CEPs) from *Lacticaseibacillus casei* strains PRA205 and 2006 were characterized at both the biochemical and genetic levels. The genomes of both *L. casei* strains included two putative CEPs genes *prtP2* and *prtR1*, but only *prtR1* was transcribed. The extracted PrtR1 proteinases were serine proteinases with optimal activity at 40 °C and pH 7.5, and were activated by Ca^2+^ ions. Interestingly, PrtR1 from *L. casei* PRA205 exhibited high residual activity at pH 4 and at 5 °C, suggesting its possible exploitation for fermented food production. The caseinolytic activity against αS1- and β-casein indicated that both PrtR1s belonged to the PI/PIII type. These PrtR1s cleaved β-casein peptide bonds preferentially when amino acid M or N was present at the P1 subsite and amino acids A and D were at the P1′ subsite. Several bioactive peptides were found to be released from PrtR1 after αs1- and β-casein hydrolysis.

## 1. Introduction

Lactic acid bacteria (LAB) are a group of microorganisms generally found in nutrient-rich environments and are commonly used in the manufacturing of fermented dairy foods. LAB are nutritionally fastidious, and their growth is dependent on the presence of external sources of nitrogen (e.g., amino acids or short peptides), since they are auxotrophic for numerous amino acids [1]. The low amounts of amino acids and peptides in milk have caused the LAB to evolve a complex proteolytic system to achieve casein hydrolysis, releasing amino acids and oligopeptides [1]. In addition to the well-studied starter LAB, some non-starter LAB (NS-LAB) also exhibit a proteolytic phenotype [2]. These NS-LAB typically colonize cheese during ripening, making an important contribution to milk protein hydrolysis, and to the formation of the texture and flavor of the fermented milk products [3].

The LAB proteolytic system involves various components, such as (1) cell-envelope proteinases (CEPs), which are responsible for the first degradation of caseins into oligopeptides; (2) a specific transport system that internalizes peptides; and (3) a wide variety of internal peptidases, such as specific endopeptidases, aminopeptidases, tri- and dipeptidases, and proline-specific peptidases [4,5].

To date, six major groups of CEPs have been described among LAB strains, including PrtP from *Lacticaseibacillus paracasei* and *Lactococcus lactis*, PrtB typical of *Lactobacillus delbrueckii* subsp. *bulgaricus*, PrtH characteristic of *Lactobacillus helveticus*, PrtS from *Streptococcus thermophilus*, PrtR found in *Lacticaseibacillus rhamnosus*, and PrtL typical of *Lactobacillus delbrueckii* subsp. *lactis* [5,6]. Moreover, CEPs have also been identified in other LAB species, such as *Lactobacillus acidophilus* and *Lactiplantibacillus plantarum* [7,8]. Most LAB are thought to have only one specific CEP, but four unique types of CEPs—namely PrtH, PrtH2, PrtH3, and PrtH4—have been characterized in *Lactobacillus helveticus* [4]. As revealed by comparative genomic analysis, the quantity of CEP genes in LAB is between 1 and 4, depending on the strain [9]. CEPs are typically synthesized in the cytoplasmic compartment in the form of pre-pro-proteinases of ~2000 amino acids [5,6]; They are organized in 6–8 functional domains comprising a signal sequence S (absent in PrtH2), a pro-domain PP, a catalytic domain PR, an insert domain I (absent in PrtR and PrtH2), the A- and B-domains, and helix domain H (only present in PrtP, PrtH, and PrtH2), a cell wall domain W, and an anchored domain AN (only present in PrtP, PrtS, and PrtR) [5,6].

In addition to the key role of CEPs in LAB growth in milk, they also play a pivotal part in developing the organoleptic properties of fermented dairy foods, and impact on the health properties of fermented dairy foods and probiotic LAB. Several studies have demonstrated that many bioactive peptides can be generated after casein hydrolysis, during both fermentation and in vitro hydrolysis by purified CEPs [6,10]. Beyond their known nutritional value, these peptides can regulate important physiological functions, since they display a plethora of activities, such as antihypertensive, antimicrobial, antithrombotic, immunomodulatory, opioid, antioxidant, and mineral-binding activities [10]. Indeed, CEPs were also able to degrade pro-inflammatory chemokines in vivo, exerting physiologically significant anti-inflammatory effects at the intestinal level [11]. Therefore, CEPs have found several applications in functional food technology [6].

The aim of this study was to characterize the genetic and biochemical features of CEPs from *Lacticaseibacillus casei* strains PRA205 and 2006—two highly proteolytic NS-LAB previously isolated from ripened Parmigiano Reggiano cheese—in order to verify their ability to produce bioactive peptides from milk proteins, along with their potential technological exploitation.

## 2. Materials and Methods

### 2.1. Strains, Species Confirmation, Culture Conditions, and Chemicals

The strains used in this study—PRA205 and 2006—were isolated from Parmigiano Reggiano cheese, and were originally identified as *L. casei* and *L. rhamnosus* [2,3]. The latter strain was attributed to *L. rhamnosus* by *tuf* gene PCR, but when we confirmed species affiliation by *mutL* gene PCR assay (Supplementary Table S1) [12], we found that strain 2006 belongs to *L. casei* (Supplementary Figures S1–S3), while we confirmed that PRA205 also belongs to *L. casei*. Species misidentification was reported between *L. rhamnosus* and *L. casei* for the *tuf* gene PCR method [12]. Strains used as controls in the experiments were *L. casei* DSMZ20011^T^, *L. rhamnosus* PRA161, and *L. rhamnosus* 0503 [2,3]. All of the reference strains were confirmed by *mutL* multiplex PCR [12]. Details of the methods used in species confirmation are reported in the Supplementary Materials. Stocks of cultures were stored frozen at −80 °C in de Man–Rogosa–Sharpe (MRS) medium (Oxoid, Basingstoke, Hampshire, UK) supplemented with 25% (*w*/*v*) glycerol. Before experimental use, all of the strains were twice propagated in MRS broth at 37 °C for 24 h, under anaerobic conditions. For the entire duration of the experiments, the reference strains were maintained in MRS medium supplemented with 7% (*w*/*v*) agar at 4 °C. All media and chemicals used in this study were purchased from Sigma-Aldrich (St. Louis, MO, USA), unless otherwise indicated. Primers and sequencing services were provided by Bio-Fab Research (Rome, Italy).

### 2.2. DNA Extraction

Genomic DNA (gDNA) was extracted from late exponential cultures grown in MRS, as described by Gala et al. [13]. Briefly, cells (1.5 mL) were centrifuged at 6000× *g* for 10 min, washed with 500 μL of TE buffer (10 mmol/L Tris-HCl, 1 mmol/L EDTA, pH 8.0), and re-suspended in 200 μL of TE buffer with glass beads (diameter 0.106 mm). Subsequently, the cell suspension was vortexed with Vortex-Genie 2 (MoBio, Jefferson City, MO, USA) for 4 min (two rounds of 2 min at the maximum speed, with 1 min in ice, and then 2 min at maximum speed). Next, 15 μL of proteinase K (20 mg/mL) was added, and the mixture was incubated at 60 °C for 1 h. After incubation, 40 μL of 20% SDS was added, and the samples were incubated at 65 °C for 15 min. After cooling at room temperature, 90 µL of refrigerated 5 mol/L potassium acetate was added, and the mixture was centrifuged at 10,000× *g* for 10 min. After performing phenol–chloroform extraction and ethanol precipitation, DNA samples were suspended in 50 μL of TE buffer. Then, the suspension was mixed with 1.5 μL of RNAse (10 mg/mL) and incubated at 37 °C for 2 h. The concentration and purity of gDNA samples were determined using a NanoDrop ND-1000 spectrophotometer (NanoDrop Technologies, Wilmington, DE, USA), while gDNA quality was evaluated by electrophoresis on 0.8% (*w*/*v*) agarose gel containing ethidium bromide (0.5 μg/mL) in 0.5X TBE buffer (45 mmol/L Tris–HCl, 45 mmol/L boric acid, and 1 mmol/L EDTA, pH 8.0). gDNA samples were diluted to 50 ng/μL in ddH_2_O and stored at −20 °C for subsequent analysis.

### 2.3. In Silico Analysis, PCR Screening, and Phylogenetic Tree Construction

The in silico search for putative *prt* genes in *L. casei* genomes was performed using the BLASTp algorithm against the NCBI database, using *L. casei* PrtP (AFJ15093.1) as the query sequence. A curated dataset containing 44 proteins from 23 strains was built, and sequences were aligned with the constraint-based alignment tool (COBALT) method [14], using the default settings (Supplementary Table S2). Conserved protein domains were analyzed via NCBI Batch CD-Search (https://www.ncbi.nlm.nih.gov/Structure/bwrpsb/bwrpsb.cgi; last access on 14 June 2021; E-Value threshold 0.01, max. hits 500) using the CDD database.

Primer pairs were designed on the conserved regions within or surrounding the catalytic domains of *prt* genes, using Primer3 [15] (Supplementary Table S3). All PCR reactions were carried out in a T100 thermal cycler (Bio-Rad, Hercules, CA, USA) with DreamTaq DNA Polymerase (Thermo Scientific, Waltham, MA, USA), following the manufacturer’s instructions. PCR products were resolved by 1.2% (*w*/*v*) agarose gel electrophoresis stained with ethidium bromide (5 μg/mL) and, when required, PCR amplicons were digested with the endonuclease *EcoR*V (Thermo Scientific, Waltham, MA, USA) in a 20 μL final volume, according to the manufacturer’s instructions. PCR products were purified with the DNA Clean & Concentrator™-5 Kit (Zymo Research, Irvine, CA, USA), and were sequenced on both strands through a DNA Sanger dideoxy sequencing process using both external and internal primers (Supplementary Table S3). Sequences were assembled in DNASTAR (DNASTAR, Inc. Madison, WI, USA) and trimmed on both ends to remove primer sequences. The nucleic acid sequences of the *prtP* and *prtR* partial genes were deposited at GenBank database under the accession numbers MZ606853 to MZ606856. Phylogenetic relationships were inferred using the gamma distribution (shape parameter = 1) model and the neighbor-joining (NJ) method. Bootstrap support values were calculated from 1000 replicates in MEGA6 [16]. All trees were visualized using the interactive tree of life (iTOL) v5.2 [17].

### 2.4. Induction of Cell-Envelope Proteinases (CEPs)

To induce the expression of CEPs, the strains were pre-cultured in 50 mL of MRS broth and incubated for 72 h at 37 °C under anaerobic conditions. After centrifugation at 10,000× *g* for 20 min at 4 °C, cells were re-suspended in physiological solution (0.9% NaCl) at a final OD600nm value of 10^10^ CFU/mL, and then spread on milk–citrate–agar (MCA) plates (4.4% re-suspended skimmed milk, 0.1% Na citrate, 0.1% yeast extract, 0.5% glucose, and 1.5% agar) in triplicate. After incubation for 48 h at 37 °C, cells were recovered from plates and re-suspended in physiological solution at a final concentration of 10^10^ CFU/mL. Samples were used for both RNA extraction and biochemical characterization.

### 2.5. RNA Extraction and RT-PCR

For RNA extraction, glassware was sterilized at 180 °C for at least 4 h to degrade RNases, and all the solutions were prepared with diethyl pyrocarbonate (DEPC)-treated water. MCA-induced cells were washed twice with DEPC-treated TE buffer (100 mmol/L Tris-HCl, 50 mmol/L EDTA, pH 8.0), and cell pellets were maintained at −80 °C until thawed with 1 mL of Tri-reagent using the Direct-zol RNA MiniPrep Kit (Zymo Research, Irvine, CA, USA). Mechanical lysis was performed using a Vortex-Genie 2 for two rounds of 20 min at the highest speed, alternated with 3 min on ice. The quantity of total RNA was measured spectrophotometrically using a NanoDrop ND-1000 (NanoDrop Technology, Wilmington, DE, USA), while the integrity was checked via denaturing gel electrophoresis on a 0.9% (*w*/*v*) agarose gel with formaldehyde (10 mL of 10× MOPS running buffer) and 18 mL of 37% formaldehyde (12 mol/L) on a pH 7.0 1× MOPS running buffer (0.4 mol/L MOPS, 1 mol/L sodium acetate, and 0.01 mol/L EDTA), after RNA treatment at 65 °C for 10 min.

Contaminating DNA was removed by treating 2 µg of RNA sample with dsDNase (Thermo Scientific, Waltham, MA, USA) at 37 °C for 2 min in a preheated thermocycler with the lid temperature adjusted to 37 °C. After being chilled on ice and briefly centrifuged, 12.5 µL treated RNA samples were used as templates for first-strand cDNA synthesis with random hexamers (Thermo Scientific, Waltham, MA, USA), using RevertAid Reverse Transcriptase (Thermo Scientific, Waltham, MA, USA), according to the manufacturer’s instructions. CEP-specific RT-PCR assays were performed with the primers reported above (Supplementary Table S3). RT-PCR of the 16S rRNA gene was carried out as previously reported, and used as the positive control [2].

### 2.6. CEP Extraction and Protein Concentration Determination

Following induction of CEPs, cells were centrifuged at 12,000× *g* for 10 min and re-suspended in 100 μL of Ca^2+^-free buffer (Tris-Cl 50 mmol/L; pH 7.5) at a final concentration of 10^10^ CFU/mL. After 30 min of incubation at 37 °C under agitation, extracts were centrifuged (12,000× *g* for 10 min), the supernatants collected, and the pellets re-suspended in the same volume of the Ca^2+^-free buffer for three additional cycles of incubation [18]. The collected supernatants containing the crude enzyme extracts were pooled and stored at −20 °C, awaiting biochemical assays. The protein concentration was assessed using the Bradford Assay Kit according to the manufacturer’s instructions (Sigma-Aldrich, St. Louis, MO, USA). The release of lactate dehydrogenase was measured in order to monitor the membrane integrity and the cell lysis during the extraction phase, as previously described [19].

### 2.7. CEPs’ Enzymatic Activity Assay

The proteinase activity of crude extracts was evaluated via a chromogenic assay. The activity was measured using the specific substrate succinyl-alanyl-alanyl-prolyl-phenylalanine-*p*-nitroanilide. The assay mixture, containing 107 μL of 50 mmol/L sodium phosphate buffer (pH 7), 56 μL of 5 mol/L NaCl, 9.5 μL of 20 mmol/L of substrate and 15 μL of extract, was incubated at 40 °C for 120 min in a covered water bath. The reaction was stopped by adding 94 μL of 80% acetic acid. The released *p*-nitroanilide was measured at 410 nm using a microplate reader. Control reactions with the substrate but without CEPs were also prepared [20]. One unit of proteinase activity was defined as the amount required to liberate 1 μmol of *p*-nitroanilide per minute. Specific activity was expressed as units of proteinase activity per mg of protein.

### 2.8. Effects of Temperature, pH, Metal Ions, and Inhibitors on CEPs’ Activity

The effect of temperature on the CEPs’ activity was measured as described above, modifying the incubation temperature. The assay was carried out at four different temperatures (5 °C, 35 °C, 40 °C, and 45 °C), at a constant optimal pH value of 7. The effect of pH on enzyme activity was evaluated by modifying the pH of the reaction buffer, keeping the temperature constant at 40 °C. Sodium acetate buffer (50 mmol/L) was utilized for reactions carried out at pH 4–6 whereas sodium phosphate buffer (50 mmol/L) was used for the reaction performed at pH 8. To analyze the influence of metal ions on proteinase activity, KCl, CaCl_2_, or MgCl_2_ was added to the reaction mixture at a final concentration of 2 mmol/L (pH 7, 40 °C). Similarly, in order to evaluate the effects of inhibitors on proteinase activity, EDTA or PMSF was added to the mix, at a final concentration of 0.5 mmol/L (pH 7, 40 °C).

### 2.9. Casein Hydrolysis

To test the caseinolytic activity of CEPs, 5 mg/mL of αS1-or β-casein solution, dissolved in sodium phosphate buffer (pH 7.0; 100 mmol/L), was mixed at a 1:1 volume ratio with the crude extracts containing 1.5 U/mL of CEP activity. The mixtures were incubated at 40 °C and, after various intervals (0, 8, 24, 30, 48, and 56 h), aliquots of samples were withdrawn and stored at −20 °C for further analysis.

### 2.10. SDS–PAGE

The casein breakdown pattern was assessed by sodium dodecyl sulfate–polyacrylamide gel electrophoresis (SDS–PAGE). Samples collected during the caseinolytic test described above were diluted in Laemmli buffer in order to load 2.5 μg of total protein per lane. Denaturation was completed by boiling the samples for 3 min. SDS–PAGE was carried out on 12% polyacrylamide gels on vertical electrophoresis cells for 1 h at 200 V. Gels were stained with the Coomassie blue staining method (0.1% Coomassie brilliant blue in 100 mL of 40% methanol, 10% glacial acetic acid, 50% H_2_O) for 1 h under stirring. Subsequently, gels were de-stained with the de-staining solution (40% methanol, 10% glacial acetic acid, 50% H_2_O) for 30 min under stirring. The de-staining step was repeated four times.

### 2.11. Identification of Peptides by Ultrahigh-Performance Liquid Chromatography/High-Resolution Mass Spectrometry (UHPLC/HRMS)

Samples collected during the casein hydrolysis assay were mixed with 1% trifluoroacetic acid at a 1:1 volume ratio and submitted to UHPLC/HRMS analysis for peptide identification. Chromatographic separation was carried out with UHPLC (UHPLC Ultimate 3000 Separation Module, Thermo Fisher Scientific, San Jose, CA, USA) equipped with a C18 column (Acquity UPLC HSS C18 Reversed Phase, 2.1 × 100 mm, 1.8 μm particle size, Waters, Milford, MA, USA). Mass spectrometry (MS) and tandem MS experiments were performed on a Q Exactive Hybrid Quadrupole-Orbitrap Mass Spectrometer (Thermo Fisher Scientific, San Jose, CA, USA). The full description of the gradient, flow rate, and MS and MS/MS parameters was reported by Martini et al. [21].

Peptide sequencing was carried out by using MASCOT (Matrix Science, Boston, MA, USA) protein identification software, with the following search parameters: enzyme, none; peptide mass tolerance, ±5 ppm; fragment mass tolerance, ±0.12 Da; variable modification, oxidation (M) and phosphorylation (ST); maximal number of post-translational modifications permitted in a single peptide, 4. The assignment procedure was confirmed by the manual verification of MS/MS spectra.

### 2.12. Identification of Bioactive Peptides

The peptides identified via mass spectrometry analysis were investigated in relation to previously identified bioactive peptides. The identification of bioactive peptides was carried out by using the Milk Bioactive Peptide Database (MBPDB)—an online database of human milk- and dairy-derived bioactive peptides [22].

### 2.13. Calculation of the Cleavage Specificity

The cleavage probability and the positive or negative influence on the cleavage of a specific amino acid in the P1 and P1′ subsites were calculated as described by Solieri et al. [23]. The amino acid residues were designated as P1 in the N-terminal direction (on the left of the sequence) and P1′ in the C-terminal direction (on the right of the sequence) from the cleaved bond. The residue P1 interacts with the subsite S1 in the enzyme active site, whereas the residue P1′ interacts with the subsite S1′ in the enzyme active site. The peptide bond cleaved by the CEPs is defined as the P1–P1′ bond.

The amino acid residue in position P1 or P1′ influences the CEP cleavage probability. If the amino acid residue *A* is in the position *n* (P1 or P1′ subsite), the cleavage probability of the P1-P1′ bond will be as follows:

(1) $$Pn=\frac{total\;amino\;acid\;A\;cleaved\;in\;position\;n}{total\;amino\;acid\;A\;in\;protein}x\;100\;$$

The mean cleavage probability was defined by the following formula:

(2) $$\%\;\bar{Pn}=\overset{20}{\underset{\#=1}{\sum }}\frac{\%Pn}{20}\;$$

The positive or negative influence of an amino acid residue *A* in the P1 and P1′ subsites was quantified by the coefficient *Kn*:

(3) $$Kn=(\%Pn/\%\bar{Pn}\bar{)-1}\;$$

*Kn* values > 0 indicate a favorable influence of the amino acid *A* in the specific subsite on the cleavage of the P1–P1′ bond, whereas *Kn* values < 0 suggest a negative effect on the cleavage.

### 2.14. Statistical Analysis

Data are shown as the mean ± standard deviation (SD) for three replicates. Univariate analysis of variance (ANOVA) with Tukey’s post hoc test was applied using GraphPad Prism 6.0 (GraphPad Software, San Diego, CA, USA). The differences were considered significant at *p* < 0.05.

## 3. Results and Discussion

### 3.1. In Silico Survey of Putative prt Genes in the L. casei Genome

Most LAB are thought to possess only one type of CEP [4], but it has been proven that more than one *prt* gene exists in the genomes of several LAB species, and that the pattern of *prt* genes is highly variable at the inter-strain level. For instance, *L. helveticus* possesses four unique types of CEP—namely *prtH1*, *prtH2*, *prtH3*, and *prtH4*—while *L. rhamnosus* CGMCC11055 possesses both *prtP* and *prtR* genes, although *prtP* was initially detected only in *L. lactis* and *L. paracasei*/*L. casei* species [4,19,24,25,26].

**Figure 1:**
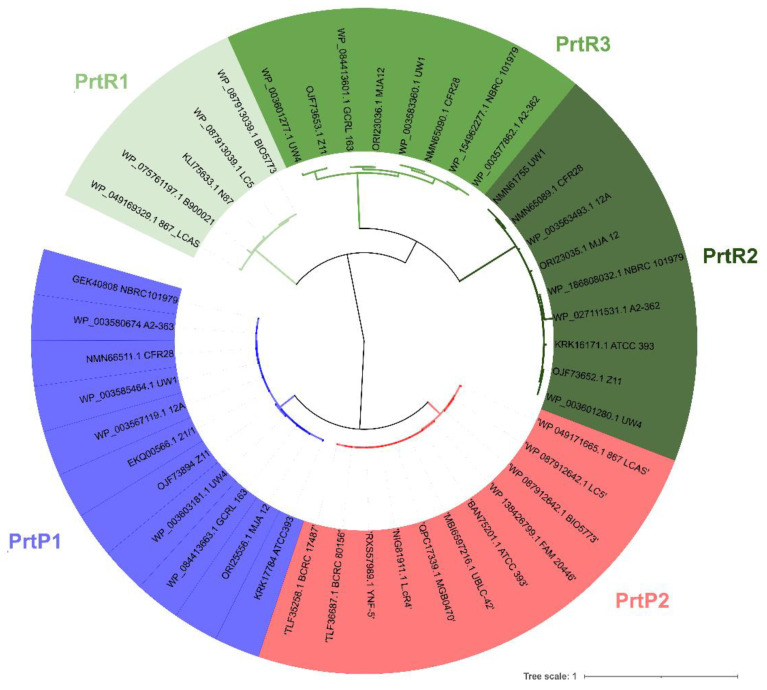
Fast minimum evolution tree of 44 putative S8 subtilisin proteases from *Lacticaseibacillus casei* genomes reported in GenBank (last access on 4 April 2021). Sequences were aligned using the COBALT tool with default parameters, while the fast minimum evolution algorithm was used to determine the distance for tree construction. Labels represent protein IDs and strain codes. The image was created using the interactive tree of life (iTOL) v5.2.

Using the *L. casei* PrtP (AFJ15093.1) amino acid sequence as a query in BLASTp search, we built a dataset consisting of 44 putative Prt-encoding genes in *L. casei*. Protein alignment with COBALT showed that 22 proteins exhibited the insert domain, and were categorized as PrtP (data not shown). The remaining 22 proteins lacked the insert domain, and were categorized as PrtR, with three different lengths of approximately 1500, 1800, and 2200 amino acids (data not shown). This result suggests that both *prtP* and *prtR* paralogs exist in several *L. casei* genomes, including in the type strain ATCC 393^T^. Clustering analysis showed two distinct clades for *L. casei* PrtP proteins, referred to as PrtP1 and PrtP2, while PrtR proteins were grouped into three different clusters, referred to as PrtR1, PrtR2, and PrtR3 (Figure 1).

“CD-search” in the Conserved Domain Database (CDD) predicted that PrtP1 and PrtP2 would share the same functional domains, except for the FIVAR domain (pfam07554)—which is present in PrtP2, but not in PrtP1—and the CHB_HEX_C domain, which is present in PrtP1, but not in PrtP2 (Supplementary Figure S4). PrtR proteins shared the subtilisin S8 family domain annotated in the Pfam database (PF00082), but mainly differed from one another at the C-terminus (Supplementary Figure S4).

### 3.2. PCR Screening and Phylogenetic Analysis

**Figure 2:**
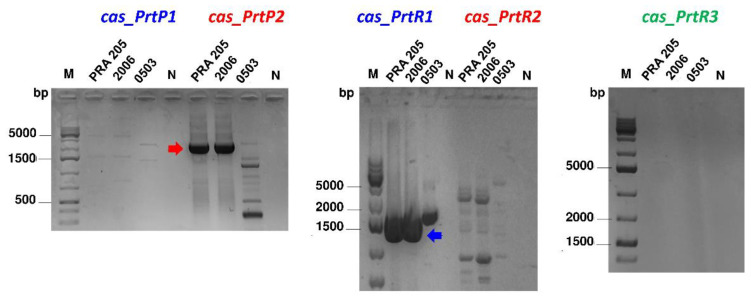
Screening of *prtP* and *prtR* genes in *Lacticaseibacillus casei* PRA205 and 2006. *Lacticaseibacillus rhamnosus* 0503 was used as a control to demonstrate the species-specificity of primer pairs. Arrows indicate expected length values. M: GeneRuler 1 kbp Plus DNA Ladders (Thermo Scientific, Waltham, MA, USA); N: negative control (PCR mixture without any DNA template).

Based on the survey of putative *prt* genes in 44 *L. casei* genomes, CEP-specific primer pairs were designed across the putative active domains of five different Prt-encoding genes, and were used to assess the type and distribution of *prtP* and *prtR* paralogs in *L. casei* PRA205 and 2006. Both strains were positive for PrtP2- and PrtR1-specific PCR assays, as they exhibited two bands with the expected lengths of approximately 2630 bp and 1723 bp, respectively, while no PCR products were obtained with the primer pairs targeting the *prtP1*, *prtR2*, and *prtR3* genes (Figure 2). This pattern of *prt* genes resembled the one we found in the genome of *L. casei* LC5, used as the reference strain in the subsequent in silico analyses. Recently, Asahina et al. [27] also identified both *prtR* and *prtP* genes in a highly proteolytic *L. paracasei* strain used as an adjunct starter in Gouda-type cheese production, whilst Vukotić et al. [26] identified three distinct proteinase genes—two prtP-type genes and one prtR-type—in *Lacticaseibacillus zeae* LMG17315. Considering the importance of the proteolytic system in assuring survival in highly protein-rich environments, the observed diversity of CEPs in mesophilic lactobacilli could arise from horizontal gene transfer, since the gain and loss of genes is a driving force in the LAB genome’s adaptive evolution [28].

To exclude the possibility that putative SNPs prevented the annealing of *prtP1*-specific primers, an additional primer set was designed on the conserved regions of the *prtP1* and *prtP2* genes. Restriction analysis of the resulting PCR amplicons with the diagnostic endonuclease *EcoR*V confirmed the presence of the *prtP2* gene (Supplementary Figure S5).

**Figure 3:**
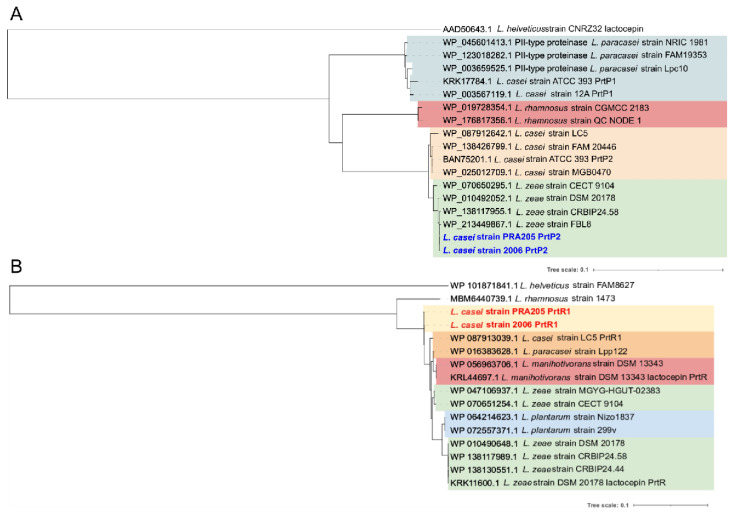
Phylogenetic trees of PrtP2 (**A**) and PrtR1 (**B**) amino acid partial sequences. The evolutionary histories were inferred using the neighbor-joining method. The percentage of replicate trees in which the associated taxa clustered together in the bootstrap test (1000 replicates) are shown next to the branches (values > 0.5). The evolutionary distances were computed using the Poisson correction method, and are given as the number of amino acid substitutions per site. The rate variation between sites was modelled with a gamma distribution (shape parameter = 1). Evolutionary analyses were conducted in MEGA6.

We sequenced the *prtP2* and *prtR1* PCR amplicons from *L. casei* PRA205 and 2006 for comparative analysis. A BLASTn search showed that the PRA205 and 2006 *prtP2* nucleotide sequences were 93.75 and 93.71% identical to the *L. casei* ATCC 393^T^ *prtP2* sequence, respectively, and even more identical (>99%) to the S8-peptidase-encoding genes in the recently released genomes of *L. zeae* strains FBL8 and CECT 9104. *L. zeae* is a recently restored species closely related to *L. casei*, and is indistinguishable from *L. casei* based on 16S rRNA gene sequencing [29,30]. BLASTx results using the predicted protein sequences revealed that the most similar protein to PRA205 and 2006 PrtP2 was WP_213449867.1 (*L. zeae* strain FBL8; 99.81%). Furthermore, PRA205 and 2006 PrtP2 partial proteins diverged from *L. casei* LC5 PrtP2 due to 13 substitutions, namely, E120K, N122S, V143A, D229A, T237A, S454R, T551A, D652A, A771T, R776G, S837G, K860R, N874S (Supplementary Figure S6). Phylogenetic analysis showed that putative PrtPs from *L. zeae* strains grouped in a homogeneous cluster divergent from *L. casei* homologs, and placed PRA205 and 2006 PrtP2 from strains more closely related to *L. zeae* than to *L. casei* ATCC 393^T^ and LC5 PrtP2 proteins (Figure 3A).

The *prtR1* nucleotide partial sequences in *L. casei* PRA205 and 2006 were 94.98, 93.07, and 77.83% identical to the *L. casei* LC5, *L. zeae* 1934, and *L. rhamnosus prtR1* genes, respectively. The predicted partial PrtR1 proteins showed six substitutions—namely V539I, T571S, S896T, A946T, V923I, and S946T—compared with LC5 PrtR1 (Supplementary Figure S7). The phylogenetic tree revealed that PrtR1 protein sequences from *L. casei*, *L. paracasei*, and *L. zeae* formed a heterogeneous group together with plasmid-encoded PrtR1 proteins from *Lacticaseibacillus manihotivorans* and *Lactiplantibacillus plantarum* (Figure 3B).

Proteinase genes (*prtP* and *prtM*) were also proven to be plasmid-encoded in *Lactococcus lactis* [31]. In *S. thermophilus*, the *prtS* gene is part of a genomic island flanked by conserved insertion sequence (IS) elements [32,33]. This mobile island has been shown to trigger gene gain and loss recombination events, which could be responsible for the huge interspecies and intraspecies variability in proteolytic activity and *prt* gene patterns observed in lactobacilli. Remarkably, PRA205 and 2006 PrtR1 partial proteins diverged from those of the *L. casei* group, and clustered separately (Figure 3B). Further experiments are required in order to establish whether the *prtR1* gene is plasmid-encoded in *L. casei* PRA205 and 2006, or whether it belongs to a genomic island flanked by IS regions.

### 3.3. CEP Extraction and prt Gene Expression Profile

Peptide-rich media generally repress proteinase activities in several lactobacilli, including *L. casei* [34,35]. Therefore, we decided to assess the CEP activities of the strains PRA205 and 2006, grown until the stationary phase on the MCA medium. Extraction of CEPs was performed using Ca^2+^-free buffer, and the specific enzyme activity was determined with the chromogenic substrate succinyl-alanyl-alanyl-prolyl-phenylalanine-*p*-nitroanilide. Strain 2006 showed a specific CEP activity of 11.34 ± 0.45 U/mg, whereas for PRA205 it was 3.73 ± 0.03 U/mg. A lactate dehydrogenase activity assay was performed in order to check the membrane integrity during the extraction process. In the crude extracts, the LDH activity was undetectable (data not shown), proving that the proteolytic activity reported was due to the action of CEPs rather than intracellular peptidases. However, we cannot exclude the possible occurrence of cell-wall-associated peptidases. The presence of cell-wall aminopeptidases has been reported for *L. lactis*, *L. helveticus*, and *Streptococcus cremoris* [36,37,38]. However, El Soda et al. [39] did not detect any aminopeptidase, dipeptidase, or carboxypeptidase activity in the cell-wall-associated proteinase fraction obtained from *L. casei*.

**Figure 4:**
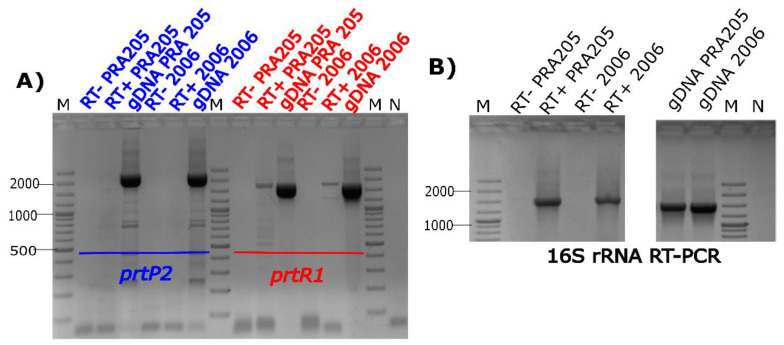
RT-PCR assay targeting the *prtP2* and *prtR1* genes in *Lacticaseibacillus casei* PRA205 and 2006 cells grown on MCA medium. The figure depicts amplified cDNAs generated with *prtP2* and *prtR1* gene (**A**)- and 16S rRNA (**B**)-specific primers from PRA205 and 2006 cells harvested in stationary phase from MCA medium; ± indicate reverse-transcription-positive and -negative controls. The 16S rRNA gene was used as a housekeeping gene. gDNA amplification was used as a positive PCR control. Abbreviations—M: molecular weight marker; N: negative control.

To qualitative establish which *prt* gene is actively transcribed in MCA-grown cells, we carried out an RT-PCR assay. Figure 4A,B show that both strains (PRA205 and 2006) transcribed *prtR1*, but not *prtP2*, suggesting that the CEP activity detected above mainly came from PrtR1 proteinase when the strains were grown on MCA medium. This result differed from that found in *L. rhamnosus* strain CGMCC11055, which has both *prtP* and *prtR* genes, but only *prtP* gene transcription was detected when grown in a synthetic medium [19]. Moreover, the results are in disagreement with those reported by Pastar et al. [40], which showed that PrtR from *L. rhamnosus* EN1 was not released following incubation with Ca^2+^-free buffer.

### 3.4. Biochemical Characterization of L. casei PRA205 and 2006 Cell-Envelope Proteinases’ PrtR1

**Figure 5:**
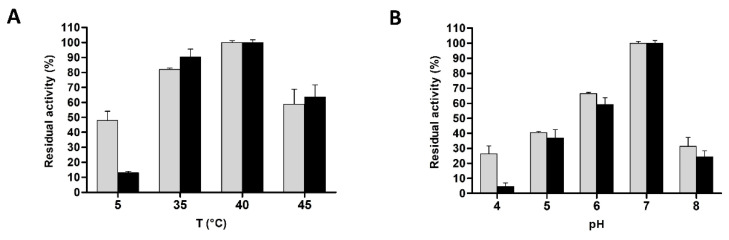
Influence of temperature (**A**) and pH (**B**) on *Lacticaseibacillus casei* PRA205 and *Lacticaseibacillus casei* 2006 PrtR1 activity. Grey bars refer to *L. casei* PRA205, whereas black bars refer to *L. casei* 2006. Results are expressed as the percentage of residual activity, set as 100% of the value at pH 7 and 40 °C. PrtR1 activity was assayed by using the specific substrate succinyl-alanyl-alanyl-prolyl-phenylalanine-p-nitroanilide. Values are the mean of three assay replications ± standard deviation (SD).

The proteolytic activity of PrtR1 was analyzed by modifying the incubation temperature and the pH of the reaction buffer in order to determine the best conditions of substrate hydrolysis. The PrtR1 extracted from each strain displayed the highest activity at 40 °C, and the enzymatic activity at 40 °C was taken as the reference (100%) for the calculation of residual (%) activity (Figure 5A). A slight decrease in the PrtR1 catalytic activity was observed at 35 °C, whereas at 5 °C the residual activity rapidly decreased for PrtR1 extracted from *L. casei* 2006, whereas the proteinase extracted from *L. casei* PRA205 retained 48% of its activity. The high proteolytic activity at 5 °C seems to be a peculiar feature of *L. casei* PRA205 PrtR1, since the majority of previously characterized CEPs almost completely lost their activity at temperatures around 20 °C [7,8,19,41]. The effect of pH on the enzyme activity was analyzed by varying the pH of the reaction buffer from 4 to 8, at a constant temperature of 40 °C (Figure 5B). All of the crude extracts achieved the highest activity at an optimal pH value of 7, which was used as the reference (100%) for the calculation of residual (%) activity. In particular, the residual activity gradually decreased at pH below and above 7 for both strains. However, when the PrtR1 activity was tested at pH 4, proteinase extracted from *L. casei* PRA205 showed a significantly higher residual proteolytic activity compared to that extracted from *L. casei* 2006.

This property could be of paramount importance because it suggests a possible exploitation of *L. casei* PRA205 in the production of bioactive peptides in fermented dairy foods such as cheese, yogurt, and fermented milk, which are characterized by low pH values. This observation could also explain the fact that *L. casei* PRA205 whole cells presented higher proteolytic activity in fermented milk than *L. casei* 2006 whole cells, despite the latter strain showing the highest CEP specific activity [2,42]. Moreover, the fact that *L. casei* PRA205 PrtR1 retained almost 50% of its activity at low temperatures suggests that the enzyme might also be active during the cold storage of fermented dairy foods, promoting the continuous release of bioactive peptides from caseins during cold storage.

The proteolytic activity of both PrtR1s was inhibited by K^+^ (71.2 ± 1.5% and 67.0 ± 6.1% of residual activity for *L. casei* PRA205 and *L. casei* 2006 PrtR1, respectively) and Mg^2+^ (80.9 ± 2.4% and 70.5 ± 6.4% of residual activity for *L. casei* PRA205 and *L. casei* 2006 PrtR1, respectively) ions when added at 2 mmol/L, and vice versa—PrtR1 activity was enhanced by 2 mmol/L Ca^2+^ ions (143.5 ± 5.3% and 183.0 ± 2% of residual activity for *L. casei* PRA205 and *L. casei* 2006 PrtR1, respectively), as reported for numerous other extracted CEPs [6]. Indeed, the activity of both PrtR1s was almost completely abolished by 1 mmol/L PMSF (86.5% and 99.9% of inhibition for *L. casei* PRA205 and *L. casei* 2006 PrtR1, respectively), suggesting that both PrtR1s were members of the serine proteinase family. The chelator EDTA at 1 mmol/L concentration also inhibited the PrtR1 activity (31.5% and 39.2% of inhibition for *L. casei* PRA205 and *L. casei* 2006 PrtR1, respectively), indicating that cations (i.e., Ca^2+^ ions) are required for their activity.

### 3.5. Caseinolytic Specificity of Extracted PrtR1, and PeptidomicsAnalysis of Casein Hydrolysates

**Figure 6:**
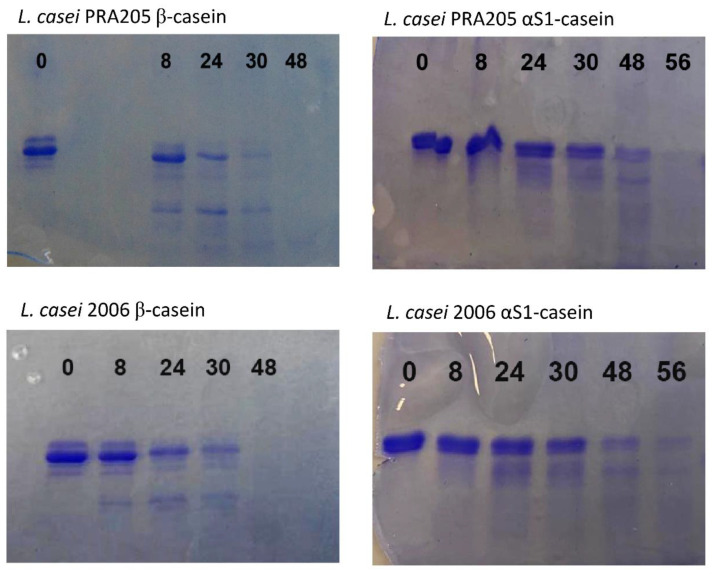
Time-course hydrolysis of β-casein and αS1-casein by PrtR1 extracted from *Lacticaseibacillus casei* PRA205 and *Lacticaseibacillus casei* 2006. β-casein or αS1-casein was added to the crude extracts containing PrtR1, and samples were taken immediately after addition and at 8, 24, 30, 48, and 56 h.

The proteolytic activity of PrtR1 was assessed against αS1- and β-caseins as substrates. Aliquots of samples were taken after 0, 8, 24, 30, 48, and 56 h, and analyzed via SDS–PAGE and mass spectrometry experiments. As shown in Figure 6, both of the extracted PrtR1s completely degraded β-casein after 48 h of incubation under the optimal conditions, while αS1-casein was hydrolyzed at a lower rate, with the protein bands still present after 56 h (Figure 6).

Based on the specific hydrolysis patterns of the αS1-, β-, and κ-caseins, CEPs were classified in three different types: CEPs belonging to the PI type primarily hydrolyze β-casein, which is cleaved into more than 100 different oligopeptides, ranging from 4 to 30 amino acid residues [6]. PIII-type CEPs can hydrolyze αS1-, β-, and κ-caseins equally well [6]. There is also a mixed CEP type named PI/PIII that cleaves β-casein in a similar manner to PI-type CEPs, and is also able to cleave αS1-casein, to a lesser extent, [10]. The above reported results indicated that β-casein was the preferential substrate over αS1-casein, suggesting that PrtR1 extracted from *L. casei* PRA205 and *L. casei* 2006 belonged to the PI/PIII type. These results are inconsistent with the findings of Kojic et al. [18], who characterized a PI-type CEP in *L. casei* HN14, while they are consistent with the mixed PI/PIII-type CEP isolated from *L. casei* IFLP 731 [41].

Samples collected during the casein hydrolysis assay were submitted to UHPLC/HRMS analysis for peptide identification in order to study the casein breakdown patterns produced by the PrtR1 extracted from the selected strains. The full list of identified peptides at the different time points for the both β- and αS1-caseins, along with the mass spectrometry data, are reported in Supplementary Tables S4–S7.

Crude extracts were incubated with β-casein for up to 48 h, and the peptidomic analysis revealed that a total of 116 and 119 peptides were released by PrtR1 extracted from *L. casei* PRA205 and *L. casei* 2006, respectively (Supplementary Tables S4 and S6). In the αS1-casein hydrolysis, samples were incubated for up to 56 h, and at the end of the incubation time a total of 102 and 124 peptides were released by PrtR1 extracted from *L. casei* PRA205 and *L. casei* 2006, respectively (Supplementary Tables S5 and S7). The number of identified peptides released from β-casein constantly increased during hydrolysis for both PrtR1s (Supplementary Tables S4 and S6, and Supplementary Figure S8A), whereas, in the case of αS1-casein, the number of peptides peaked after 48 h of incubation (Supplementary Tables S5 and S7 and Supplementary Figure S8B). As reported in the Venn diagrams (Supplementary Figure S9), 57.7% of peptides released from β-casein and 61.4% of peptides released from αS1-casein were commonly found in the hydrolysates obtained from the two PrtR1s, suggesting a similar cleavage specificity.

### 3.6. Analysis of the β-Casein Cleavage Site Specificity

**Figure 7:**
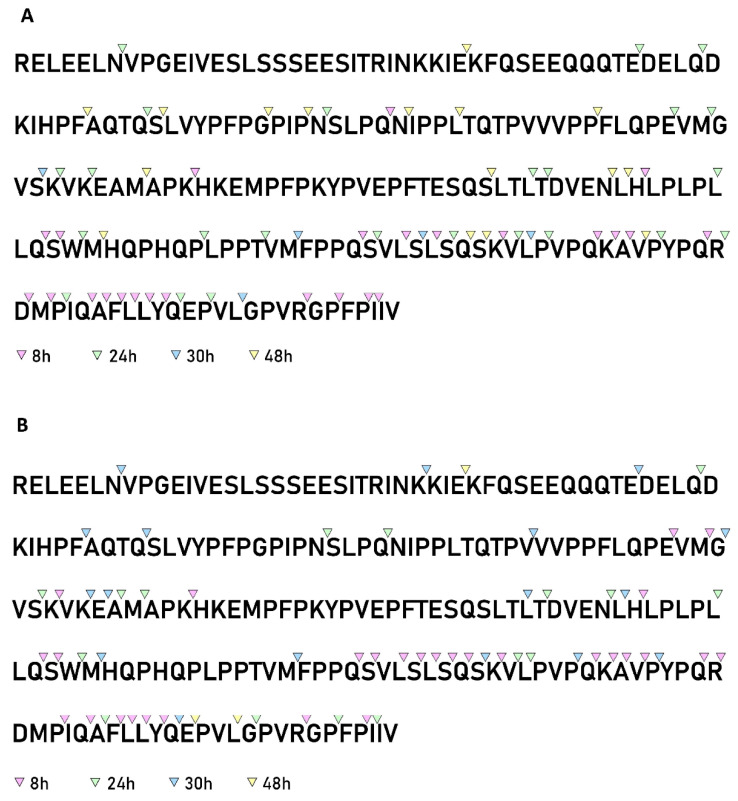
Distribution of the cleavage sites identified in the primary sequences of β-casein by PrtR1 from *Lacticaseibacillus casei* PRA205 and *Lacticaseibacillus casei* 2006. (**A**) β-casein hydrolysis by *Lacticaseibacillus casei* PRA205, and cleavage sites identified after 8, 24, 30, and 48 h of hydrolysis. (**B**) β-casein hydrolysis by *Lacticaseibacillus casei* 2006, and cleavage sites identified after 8, 24, 30 and 48 h of hydrolysis.

The analysis of the β-casein cleavage site specificity revealed the presence of 63 and 66 different cleavage sites in samples hydrolyzed by PrtR1 extracted from *L. casei* PRA205 and *L. casei* 2006, respectively (Figure 7A,B); these represented 30.3% and 31.7% of all peptide bonds present in the proteins, respectively. A total of 54 cleavage sites were in common between the two PrtR1s, representing 85.7 and 81.8% of the total sites found in *L. casei* PRA205 and *L. casei* 2006 CEPs, respectively, suggesting that these CEPs had almost the same cleavage specificity.

The time-course analysis indicated that the hydrolysis of β-casein began at the hydrophobic C-terminal region. After 8 h of reaction, 80% and 76% of the cleavage sites produced by *L. casei* PRA205 and *L. casei* 2006 PrtR1, respectively, were located between residue 161 and the C-terminal amino acid V at position 209. At this point in time, no cleavage sites were found in the N-terminal region between residues 1 and 72 or in the central region situated between the amino acids at positions 106 and 134. Furthermore, considering the long region between amino acids 1 and 134, only two and four cleavage sites were detected in the *L. casei* PRA205 and *L. casei* 2006 PrtR1 hydrolysates, respectively. After 24 h of hydrolysis, the majority (59% for *L. casei* PRA205 PrtR1 and 62% for *L. casei* 2006 PrtR1) of the cleavage sites were still in the C-terminal region. At the end of the hydrolysis, the cleavage sites were distributed throughout the entire β-casein sequence for both of the PrtR1s; however, no cleavage sites were identified in the polyphosphorylated region between residues 8 and 28. It is worth noting that the majority of the previously characterized lactobacilli proteinases have a proven preference for hydrolyzing the C-terminal region of β-casein [6,43].

Furthermore, the cleavage probability at subsites P1 and P1′, along with the *Kn* coefficients, which measure the positive or negative effects of amino acids on the P1–P1′ cleavage probability, were calculated (Table 1).

PrtR1 extracted from both *L. casei* strains showed a marked preference for the amino acid M, for polar uncharged amino acids (S, Q, and N), and for positively charged amino acids (R and K) in the P1 position. In particular, the amino acids M and N showed the highest *Kn* values for both PrtR1s, therefore exerting the strongest positive effect on cleavage probability at the P1 subsite. The only difference between the two PrtR1s was the strong positive effect of amino acid A on PrtR1 extracted from *L. casei* 2006. In position P1, the amino acids I, T, P, V, and H, as well as the negatively charged (D and E) and aromatic (F and Y) amino acids, exhibited a strong negative effect on cleavage probability for both of the extracted PrtR1s.

The strongest positive effect on cleavage probability at subsite P1′ was found for the small aliphatic amino acids A and G, as well as the amino acids H and D, for both of the extracted PrtR1s. In both cases, the amino acids D and A showed the highest *Kn* values. The strongest negative effect at position P1′ was exhibited by the polar uncharged amino acids (especially P, Q, and T) and by the amino acid E. Some of the identified preferred amino acids have already been reported in previous studies; for example, the positive effect of amino acid Q in position P1 has been already described for CEPs characterized from *L. delbrueckii* CRL581, *L. helveticus* BGRA43, *L. paracasei* BGHN14, *L. rhamnosus* BGT10 and PRA331, and *Lc. lactis* NCDO763 [20,23,43,44]. The preference for this amino acid at the P1 subsite seems to be a common feature among the CEPs extracted from lactobacilli belonging to different species. In addition, the preference for N and M at position P1 has already been described for *L. lactis* NCDO763 and *L. rhamnosus* PRA331, respectively [23,44]. To the best of our knowledge, the strong positive effects exerted by the positively charged amino acids (R and K) in the P1 position and by the residue D in the P1′ position have never been reported in the CEPs of LAB. The profile of cleavage-site specificity was slightly distinct from that previously reported for *L. casei* PRA205 [23]. These differences may be related to different experimental conditions, e.g., hydrolysis carried out with PRA205 whole cells and milk.

### 3.7. Analysis of the αS1-Casein Cleavage Site Specificity

**Figure 8:**
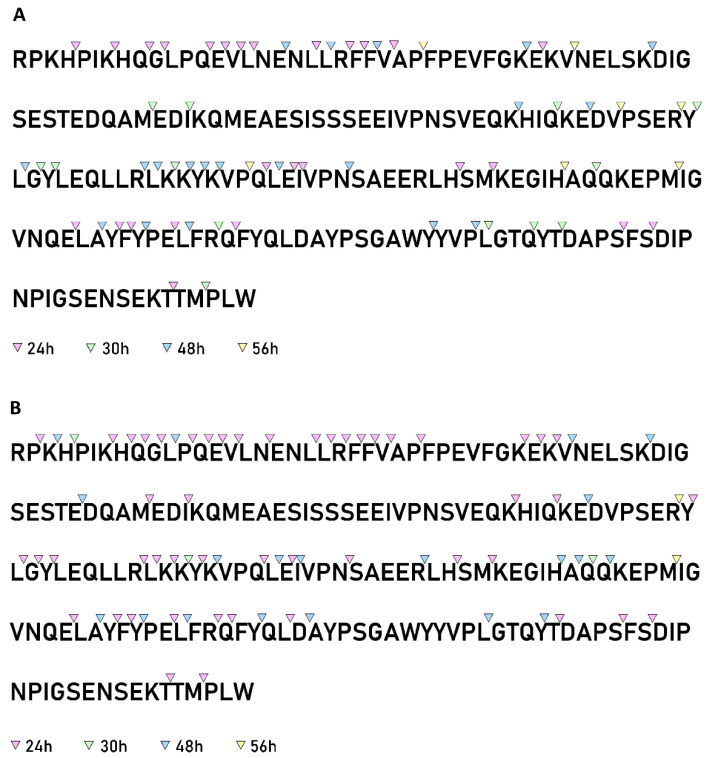
Distribution of the cleavage sites identified in the primary sequences of αS1-casein by PrtR1 from *Lacticaseibacillus casei* PRA205 and *Lacticaseibacillus casei* 2006. (**A**) αS1-casein hydrolysis by *Lacticaseibacillus casei* PRA205, and cleavage sites identified after 24, 30, 48, and 56 h of hydrolysis. (**B**) αS1-casein hydrolysis by *Lacticaseibacillus casei* 2006, and cleavage sites identified after 24, 30, 48, and 56 h of hydrolysis.

Unlike β-casein, the hydrolysis of αS1-casein started at the N-terminal part of the protein. After 24 h of incubation, 50% and 45% of the cleavage sites were located in the sequence between residues 1 and 36 for PrtR1 extracted from *L. casei* PRA205 and *L. casei* 2006, respectively (Figure 8A,B). Most of these cleavage sites were positioned in the fragment 1–23. Additional preferred cleavage sites at the beginning of the hydrolysis occurred in the sequence 90–110 for *L. casei* 2006 PrtR1, and in sequence 140–160 for both extracted CEPs. At the end of hydrolysis, the cleavage sites were mainly concentrated in the fragments 1–40 and 90–160, as already reported for the majority of characterized CEPs [6].

### 3.8. Identification of Bioactive Peptides Using the Milk Bioactive Peptide Database

The identification of bioactive peptides released by PrtR1 was carried out by searching all of the peptides found in the mass spectrometry analysis experiments against the Milk Bioactive Peptide Database (MBPDB). The hydrolysis of β-casein by PrtR1 of *L. casei* PRA205 and *L. casei* 2006 produced 14 and 17 functional peptides previously demonstrated to have several bioactivities, respectively (Table 2).

The PrtR1 of both *L. casei* strains commonly released a total of 13 bioactive peptides, whereas 4 were uniquely produced by *L. casei* 2006 PrtR1 and 1 by *L. casei* PRA205 PrtR1. In contrast, the hydrolysis of αS1-casein by PrtR1 of *L. casei* PRA205 and *L. casei* 2006 generated 9 and 11 bioactive peptides, respectively, among which 8 were commonly released by the PrtR1 of both strains.

The identified bioactive peptides showed several functional properties, as 13 peptides were ACE inhibitors, 13 were antimicrobial, 6 were antioxidants, 5 had immunomodulatory activity, 1 showed dipeptidyl peptidase IV (DPPIV)-inhibitory activity, and 1 showed anxiolytic activity. Most of the identified bioactive peptides were tested in vitro for their bioactivity, whereas some of them also exhibited their activities in vivo. For example, the tripeptide LLY demonstrated a positive influence in vivo in Swiss albino mice against ethanol-induced oxidative stress [45]. The β-casein fragment KVLPVPQ showed antihypertensive activity in vivo in spontaneously hypertensive rats (SHRs) [10]. In addition, the bioactive peptide LYQEPVLGPVRGPFPIIV exerted its immunomodulatory activities by stimulating lymphocyte proliferation in rats [46].

With the sole exception of the αS1-casein-derived peptides LGY and RPKHPIKHQGLPQEVLNENLLRFFVAPFPEVFGKEK, all the other identified bioactive peptides were found at the end of the hydrolysis, suggesting their resistance to further hydrolysis by PrtR1 (Table S3). In addition, 16 bioactive peptides were released only in the final periods of hydrolysis.

To exert their physiological effect, bioactive peptides should be stable under gastrointestinal conditions and resistant to the hydrolysis by gastrointestinal proteases. In a recent study, it was demonstrated that most of the peptides identified after in vitro gastro-pancreatic digestion contained 1–4 proline residues near the carboxylic ends of their sequences. In addition, the simultaneous presence of DPP-IV-inhibitory peptides causes a strong decrease in the intestinal prolyl peptidases’ activity [47]. Among the identified peptides, LLYQEPVLGPVRGPFPIIV, YQEPVLGPVRGPFPIIV, and QEPVLGPVRGPFPIIV were found after simulating gastro-pancreatic digestion of homogenized yogurt; in addition, KVLPVPQ, VLPVPQK, YQEPVLGPVRGPFPIIV, LPVPQ, and EPVLGPVRGPFP were found in the human gastrointestinal tract [48].

## 4. Conclusions

In this work, two unique CEPs from the highly proteolytic strains *L. casei* PRA205 and *L. casei* 2006, previously isolated from ripened Parmigiano Reggiano cheese, were characterized. Both strains possessed two different *prt* genes in their genome—*prtP2* and *prtR1*—but the evidence collected in this study suggests that only one gene, *prtR1*, is actively transcribed in MCS-induced cells after 48 h of incubation. In both strains, the predicted protein catalytic domain sequences of PrtR1 showed six amino acid substitutions compared with the reference sequence, suggesting that these proteases were peculiar to the selected strains. In addition, PrtR1 identified in *L. casei* PRA205 and 2006 showed high similarity with PrtR1-like sequences which are plasmid-encoded in other LAB species. The presence of PrtR1 on plasmids could confer an important evolutionary advantage to these strains, but further analysis is required to establish the true gene position.

PrtR1 extracted from both strains displayed the highest activity at 40 °C, pH 7; interestingly, PrtR1 extracted from *L. casei* PRA205 retained 48% of its activity at 5 °C and showed higher activity at pH 4 than strain *L. casei* 2006. These important biotechnological features can be exploited to produce fermented dairy products with low pH and low storage temperatures, such as fermented milk and yoghurt.

Remarkably, peptidomic analysis assisted us in demonstrating that these CEPs are able to release β- and αS1-casein-derived bioactive peptides. Most of these peptides matched the sequences of previously reported bioactive peptides, and some of them were resistant to gastro-intestinal hydrolysis.

Overall, the results presented in this study provide new knowledge on the proteolytic systems of two strains belonging to the *L. casei* species, which is poorly explored in comparison with thermophilic lactobacilli or lactococci. Furthermore, PrtR1 from both strains were able to release some bioactive peptides, suggesting that *L. casei* can be a source of new proteases exploitable as enzymes for the formulation of dairy beverages or hydrolysates enriched in bioactive peptides, and with improved digestibility of milk proteins. Thereby, NS-LAB can be considered as a source of new enzymes, and the knowledge of CEPs developed in this work can be used as a tool for the selection of new proteinase-proficient strains, as well as for the improvement of functional lactobacilli performance—particularly in the *L. casei* species.

## Figures and Tables

**Table 1:** Cleavage occurrence, cleavage probability (%P), and *Kn* coefficients for *Lacticaseibacillus casei* PRA205 and *Lacticaseibacillus casei* 2006 PrtR1 on β-casein in different amino acids at the P1 and P1′subsites after 48 h of hydrolysis.

	*L. casei* PRA205 PrtR1				*L. casei* 2006 PrtR1			
Amino Acid ^1^	P1 Subsite		P1′ Subsite		P1 Subsite		P1′ Subsite	
	Number of Cleaved Bonds ^2^ (%P1 ^3^)	Kn	Number of Cleaved Bonds ^2^ (%P1′ ^3^)	Kn	Number of Cleaved Bonds ^2^ (%P1 ^3^)	Kn	Number of Cleaved Bonds ^2^ (%P1′ ^3^)	Kn
Aliphatic								
A (5)	2 (40.0)	0.08	4 (80.0)	0.86	3 (60.0)	0.69	5 (100.0)	1.42
G (5)	1 (20.0)	−0.46	3 (60.0)	0.40	2 (40.0)	0.13	3 (60.0)	0.45
V (19)	3 (15.8)	−0.58	9 (47.4)	0.10	3 (15.8)	−0.56	8 (42.1)	0.02
L (22)	10 (45.5)	0.22	10 (45.5)	0.06	9 (40.9)	0.15	8 (36.4)	−0.12
I (10)	1 (10.0)	−0.73	4 (40.0)	−0.07	1 (10.0)	−0.72	3 (30.0)	−0.27
M (6)	5 (83.3)	1.24	2 (33.3)	−0.22	4 (66.7)	0.88	2 (33.3)	−0.19
Polar un-charged								
T (9)	2 (22.2)	−0.40	2 (22.2)	−0.48	1 (11.1)	−0.69	1 (11.1)	−0.73
S (16)	8 (50.0)	0.35	7 (43.8)	0.02	7 (43.8)	0.23	7 (43.8)	0.06
Q (20)	10 (50.0)	0.35	2 (10.0)	−0.77	10 (50.0)	0.41	3 (15.0)	−0.64
N (5)	4 (80.0)	1.15	2 (40.0)	−0.07	3 (60.0)	0.69	1 (20.0)	−0.52
P (35)	8 (22.9)	−0.38	4 (11.4)	−0.73	5 (14.3)	−0.60	4 (11.4)	−0.72
Positively charged								
R (4)	2 (50.0)	0.35	1 (25.0)	−0.42	2 (50.0)	0.41	1 (25.0)	−0.40
K (11)	5 (45.5)	0.22	4 (36.4)	−0.15	6 (54.6)	0.54	5 (45.5)	0.10
H (5)	1 (20.0)	−0.46	3 (60.0)	0.40	1 (20.0)	−0.44	3 (60.0)	0.45
Negatively charged								
E (19)	3 (15.8)	−0.58	2 (10.5)	−0.76	5 (26.3)	−0.26	2 (10.5)	−0.75
D (4)	1 (25.0)	−0.33	4 (100.0)	1.33	0 (0)	−1.00	4 (100.0)	1.42
Aromatic								
F (9)	2 (22.2)	−0.40	4 (44.4)	0.03	2 (22.2)	−0.37	3 (33.3)	−0.19
Y (4)	1 (25.0)	−0.33	2 (50.0)	0.16	1 (25.0)	−0.30	2 (50.0)	0.21

^1^ One code letter was used for amino acid nomenclature. The amino acid C is not present in the sequence of β-casein, while the amino acid W was omitted from the analysis because it occurs only once in the β-casein sequence. Numbers in brackets are the number of specific residues in the protein sequence. ^2^ The cleaved bonds are reported in Figure 7. ^3^ See the Materials and Methods section for the calculation of the %P1 and %P1′ cleavage probability and the *Kn* coefficient. Amino acids with a positive impact on the cleavage probability are shown in bold.

**Table 2:** Peptides with previously demonstrated bioactivity (100% homology) identified in the β-casein and αS1-casein hydrolysates produced by PrtR1 activity of *Lacticaseibacillus casei* PRA205 and *Lacticaseibacillus casei* 2006.

Sequence	Fragment	Bioactivity	PrtR1
VVPP	β-casein f(83–86)	ACE inhibition	*L. casei* PRA205
VKEAMAPK	β-casein f(98–105)	Antioxidant Antimicrobial	*L. casei* 2006
EAMAPK	β-casein f(100–105)	Antimicrobial	*L. casei* 2006
SQSKVLPVPQ	β-casein f(166–175)	ACE inhibition	*L. casei* PRA205 *L. casei* 2006
KVLPVPQ	β-casein f(169–175)	ACE inhibition	*L. casei* 2006
VLPVPQKAVPYPQR	β-casein f(170–183)	Antimicrobial	*L. casei* PRA205 *L. casei* 2006
VLPVPQK	β-casein f(170–176)	ACE inhibition Antioxidant Antimicrobial	*L. casei* 2006
LPVPQ	β-casein f(171–175)	DPP-IV inhibition	*L. casei* PRA205 *L. casei* 2006
PYPQ	β-casein f(179–182)	Antioxidant	*L. casei* PRA205 *L. casei* 2006
RDMPIQAF	β-casein f(183–190)	ACE inhibition	*L. casei* PRA205 *L. casei* 2006
LLY	β-casein f(191–193)	Immunomodulation	*L. casei* PRA205 *L. casei* 2006
LLYQEPVLGPVRGPFPIIV	β-casein f(191–209)	ACE inhibition	*L. casei* PRA205 *L. casei* 2006
LYQEPVLGPVRGPFPIIV	β-casein f(192–209)	Immunomodulation	*L. casei* PRA205 *L. casei* 2006
YQEPVLGPVR	β-casein f(193-202)	ACE inhibition Immunomodulation	*L. casei* PRA205 *L. casei* 2006
YQEPVLGPVRGPFPI	β-casein f(193–207)	Antimicrobial	*L. casei* PRA205 *L. casei* 2006
YQEPVLGPVRGPFPIIV	β-casein f(193–209)	ACE inhibition Immunomodulation Antimicrobial	*L. casei* PRA205 *L. casei* 2006
QEPVLGPVRGPFPIIV	β-casein f(194–209)	ACE inhibition	*L. casei* PRA205 *L. casei* 2006
EPVLGPVRGPFP	β-casein f(195–206)	ACE inhibition	*L. casei* PRA205 *L. casei* 2006
RPKHPIK	αS1-casein f(1–7)	Antimicrobial	*L. casei* 2006
RPKHPIKHQ	αS1-casein f(1–9)	ACE inhibition	*L. casei* 2006
RPKHPIKHQGLPQEVLNENLLRF	αS1-casein f(1–23)	Immunomodulation Antimicrobial	*L. casei* PRA205 *L. casei* 2006
RPKHPIKHQGLPQEVLNENLLRFF	αS1-casein f(1–24)	Antimicrobial	*L. casei* PRA205 *L. casei* 2006
RPKHPIKHQGLPQEVLNENLLRFFVAPFPEVFGKEK	αS1-casein f(1–36)	Antimicrobial	*L. casei* 2006
VLNENLLR	αS1-casein f(15–22)	Antimicrobial	*L. casei* PRA205 *L. casei* 2006
YLGYLEQLLR	αS1-casein f(91–100)	Anxiolytic	*L. casei* PRA205
LGY	αS1-casein f(92–94)	ACE inhibition Antioxidant	*L. casei* PRA205 *L. casei* 2006
LGYLEQLLRL	αS1-casein f(92–101)	Antimicrobial	*L. casei* PRA205 *L. casei* 2006
YLEQLLR	αS1-casein f(94–100)	Antimicrobial	*L. casei* PRA205 *L. casei* 2006
PEL	αS1-casein f(147–149)	Antioxidant	*L. casei* PRA205 *L. casei* 2006
GTQYTDAPSFSDIPNPIGSENSEKTTMPLW	αS1-casein f(170–199)	ACE inhibition Antioxidant	*L. casei* PRA205 *L. casei* 2006

Abbreviations—ACE: angiotensin-converting enzyme; DPP-IV: dipeptidyl peptidase IV.

## Data Availability

The data presented in this study are available in here and in supplementary material.

## Supplementary Materials

The following are available online at https://www.mdpi.com/article/10.3390/biology11010139/s1: Figure S1: Phylogenetic relationships among *Lacticaseibacillus casei* species inferred by the neighbor-joining (NJ) method from partial *mutL* gene sequences; Figure S2: *mutL*-targeted PCR amplification of *L. casei* 2006 and PRA205; Figure S3: Phylogenetic tree generated from 1473 nucleotide positions of the strain 2006 16S rRNA gene; Figure S4: Representative selection of conserved domains identified in PrtP1, PrtP2, PrtR1, PrtR2, and PrtR3; Figure S5: PCR-based approaches to dissect *PrtP* variants in *Lacticaseibacillus casei* PRA205 and 2006; Figure S6: Multiple sequence alignment of PRA205 and 2006 PrtP2 partial proteins with the PrtP2 reference protein from *Lacticaseibacillus casei* LC5; Figure S7: Multiple sequence alignment of PRA205 and 2006 PrtR1 partial proteins with the Prtr1 reference protein from *Lacticaseibacillus casei* LC5; Figure S8: Number of peptides identified in β-casein and αS1-casein hydrolysates at the different time points; Figure S9: Venn diagram displaying differences between peptides produced after β-casein (**A**) or aS1-casein (**B**) hydrolysis by *Lacticaseibacillus casei* PRA205 and 2006; Table S1: Primers used in *mutL*-gene-targeted multiplex PCR; Table S2: Dataset containing *L. casei* genomes’ accession numbers, putative *prt* gene coordinates (in brackets), and protein IDs; Table S3: Primer sequences, their targets, and the annealing temperatures used for *Lacticaseibacillus casei* prtP and prtR gene screening and sequencing; Table S4: List of peptides identified at the different time points during hydrolysis of β-casein with PrtR1 from *Lacticaseibacillus casei* PRA205; Table S5: List of peptides identified at the different time points during hydrolysis of αS1-casein with PrtR1 from *Lacticaseibacillus casei* PRA205; Table S6: List of peptides identified at the different time points during hydrolysis of β-casein with PrtR1 from *Lacticaseibacillus casei* 2006; Table S7: List of peptides identified at the different time points during hydrolysis of αS1-casein with PrtR1 from *Lacticaseibacillus casei* 2006.
