# Supplementary material for: Characterization of Cell-Envelope Proteinases from Two Lacticaseibacillus casei Strains Isolated from Parmigiano Reggiano Cheese
Source: Biology (Basel). 2022 Jan 14;11(1):139. doi: 10.3390/biology11010139 (PMC8773131; doi:10.3390/biology11010139)
Supplement: Supplementary file 1 [file biology-11-00139-s001.zip › biology-1479218-supplementary.pdf]

biology

MDPI

Article

Characterization of cell-envelope proteinases from two *Lacticaseibacillus casei* strains isolated from Parmigiano Reggiano cheese

Lisa Solieri [1], Laura Sola [1], Amanda Vaccalluzzo [2], Cinzia Lucia Randazzo [2,3], Serena Martini [1] and Davide Tagliazucchi [1*]

[1] Department of Life Sciences, University of Modena and Reggio Emilia, Via Amendola, 2 - Pad. Besta, 42100 Reggio Emilia, Italy; lisa.solieri@unimore.it; laura.sola@unimore.it; serena.martini@unimore.it; davide.tagliazucchi@unimore.it

[2] Department of Agriculture, Food and Environment, University of Catania, via Santa Sofia, 100, 95123 Catania, Italy; amanda.vaccalluzzo@unict.it; cranda@unict.it

[3] ProBioEtna srl, Spin off University of Catania, via Santa Sofia, 100, 95123 Catania, Italy; cranda@unict.it

* Correspondence: davide.tagliazucchi@unimore.it; Tel.: +39 0522 522060

Citation: Solieri, L.; Sola, L.; Vaccalluzzo, A.; Randazzo, C.L.; Martini, S.; Tagliazucchi, D. Characterization of Cell-Envelope Proteinases from Two *Lacticaseibacillus casei* Strains Isolated from Parmigiano Reggiano Cheese. *Biology* **2022**, *11*, 139. https://doi.org/10.3390/biology11010139

Academic Editor: Rosa Siciliano

Received: 9 November 2021
Accepted: 12 January 2022
Published: 14 January 2022

Publisher's Note: MDPI stays neutral with regard to jurisdictional claims in published maps and institutional affiliations.

Copyright: © 2022 by the authors. Licensee MDPI, Basel, Switzerland. This article is an open access article distributed under the terms and conditions of the Creative Commons Attribution (CC BY) license (https://creativecommons.org/licenses/by/4.0/).

Simple Summary: Lactic acid bacteria are nutritionally fastidious microorganisms typically used in the production of fermented dairy foods. The lactic acid bacteria proteolytic system is crucial for their growth in milk and play a paramount role in developing the organoleptic and healthy properties of fermented dairy foods. Cell-envelope proteinases are the first component of this system, responsible for the degradation of caseins into short peptides. In the present work, the cell-envelope proteinases of two highly-proteolytic *Lacticaseibacillus casei* strains, previously isolated from ripened Parmigiano Reggiano cheese, were characterized from a genetic and biochemical point-of-view. Two different *prt* genes existed in the genome of both the strains but only one, named PrtR1, was expressed. PrtR1 proteins extracted from both the strains displayed the highest activity at 40°C and pH 7. Interestingly, PrtR1 extracted from *Lacticaseibacillus casei* PRA205 retained some residual activity at 5°C and at pH 4. These important bio-technological features can be exploited in the production of fermented dairy products. Peptidomics analysis revealed that both the proteinases were able to release β- and αS1-casein-derived bioactive peptides, suggesting that *Lacticaseibacillus casei* can be a source of new proteinases that can be exploited for the formulation of dairy beverages or hydrolysates enriched in bioactive peptides.

Abstract: In the present work, two cell-envelope proteinases (CEPs) from *Lacticaseibacillus casei* strains PRA205 and 2006 have been characterized at both the biochemical and genetic levels. The genomes of both the *L. casei* strains included two putative CEPs genes *prtP2* and *prtR1*, but only *prtR1* was transcribed. The extracted PrtR1 proteinases were serine proteinase with optimal activity at 40 °C and pH 7.5 and activated by Ca^{2+} ions. Interestingly, PrtR1 from *L. casei* PRA205 exhibited high residual activity at pH 4 and at 5 °C, suggesting its possible exploitation for fermented food production. The caseinolytic activity against αS1- and β-casein indicated that both the PrtR1 belong to PI/PIII type. These PrtR1 cleaved β-casein peptide bonds preferentially when amino acid M or N were present at the P1 sub-site and amino acid A and D at the P1′ sub-site. Several bioactive peptides were found to be released from PrtR1 after αs1- and β-casein hydrolysis.

Keywords: lactic acid bacteria; fermented food; functional food; bioactive peptides; protease; peptidomics

1. Supplementary Materials and Methods

1.1 mutL gene-targeted multiplexPCR assay and 16S rRNa gene re-sequencing

Species attribution was confirmed by *mutL* gene-targeted multiplex PCR assay, according to Bottari et al. [1]. Primers used were listed in Table S1. Briefly, PCR amplification reactions were carried out in a final volume of 20 μL containing 1X DreamTaq Green Buffer (containing 2 mM $MgCl_2$), 200 μM of each dNTP, template DNA, 0.5 U of DreamTaq Green DNA polymerase and 0.25 μM of each primer.

Table S1 Primers used in *mutL* gene-targeted multiplex PCR

Primer name	Sequence (5′->3′)	Species target	Expected length (bp)
CZfor	CAGCGCTGGTGGAAGACTTG	L. casei/L. zeae	666
PC2a	GGATTGGGTTTTGCGTGATGGTCGC	L. paracasei	253
RHfor	GACTTCTCAACCAGCAGCGCAGA	L. rhamnosus	801
CPRrev	TGCATTTCCCCGCTTTCATGACT	unique	na

Abbreviation: na, not applicable

The cycling parameters of *mutL* gene-targeted multiplex PCR assay included initial denaturation at 94 °C for 5 min; 35 cycles of 94 °C for 30 s, annealing at 68 °C for 30 s, and extension at 72 °C for 45 s; final extension at 72 °C for 5 min. Finally, aliquots of 10 μL PCR products were detected on 1.8% (w/v) agarose gel containing ethidium bromide (0.5 mg/mL) in 0.5X TBE buffer at constant voltage of 90 V for 1 h at room temperature. As molecular-weight standard was used GeneRuler 100 bp DNA ladder (Thermo Fisher Scientific, Waltham, MA). References strains were *L. rhamnosus* 0503 [2], *L. rhamnosus* PRA161 [3], *L. casei* PRA041 [3], and *L. casei* DSM2011^T.

The 16S rRNA gene resequencing of strain 2006 was carried out as reported by Tagliazucchi et al [2] with external primer 27f [4] and the internal primer WLAB2 (5′-TCGAATTAAACCACATGCTCCA-3′) [5]. The *mutL* and 16S rRNA gene sequences of almost all reference strains of *L. casei*, *L. zeae*, *L. paracasei*, and *L. rhamnosus* were obtained directly from the GenBank database according to the GenBank/ENA/DDBJ accession numbers. Sequences were aligned by ClustalW [6]. Phylogenetic trees were reconstructed using the neighbour-joining method (with the Kimura 2-parameter method) [7,8]. The bootstrap analysis was performed based on 1000 replicates [9]. The MEGA 6 package was used for all phylogenetic analyses [10].

All trees were visualized using the interactive tree of life (iTOL) v5.2 [11]. All amplification reactions were performed with a T100™ thermal cycler.

1.2 Prt proteins database construction and alignment

The Constraint-Based Alignment Tool method (Cobalt) was used with the default settings [12] to align a non-redundant database of 44 Prt proteins annotated in *L. casei* genomes. The Cobalt tool anchors the alignment using constraints derived from the conserved domain database (CDD) and PROSITE protein-motif database so that conserved residues of Prt proteins were accurately aligned. Prt database and cobalt output were reported in Supplementary Table S2 and Figure S2, respectively.

Proteins alignments were carried out with Clustal Omega online tool (https://www.ebi.ac.uk/Tools/msa/clustalo/]) and visualized with Jalview [13].

Table S2. Dataset containing *L. casei* genomes accession numbers, putative *prt* genes coordinates (in brackets), and protein IDs.

Strain	*prtP1*	*prtP2*	*prtR1*	*prtR2*	*prtR3*
**ATCC 393**	AZCO01000003.1 (75198..80906); KRK17784.1	AP012544.1 (2174096..2179978); BAN75201.1		AZCO01000017.1: (14519..20047); KRK16171.1	
**LC5**		NZ_CP017065.1 (2282143-2288025); WP_087912642.1	NZ_CP017065.1 (2903816..2908414); WP_087913039.1		
**MGB0470**		CP064303.1 (1449026..1454908); QPC17339.1			
**12A**	NZ_CP006690.1 (2245339..2239631); WP_003567119.1			NZ_CP006690.1 (c522337..516908);WP_003563493.1	
**N87**			LCUN01000013.1 (514473..519071); KLI75633.1		
**BIO5773**		NZ_WBOC01000001.1 (403476..409358); WP_087912642.1	NZ_WBOC01000004.1 (8424..13022); WP_087913039.1		
**L.cR4**		JAAQWB010000001.1 (110060..115942); NIG81911.1			
**CRF28**	JDWL01000024.1 (4256..9964); NMN66511.1			JDWL01000006.1 (19052..24481); NMN65089.1	JDWL01000006.1 (25023..31721); NMN65090.1
**GCRL 163**	NZ_MODT01000052.1 (29810..35518); WP_084413663.1				NZ_MODT01000038.1 (700..7398); WP_084413601.1
**MJA 12**	NZ_MODT01000052.1 (29810..35518); ORI25526.1			MODS01000094.1 (31287..36716); ORI23035.1	MODS01000094.1 (37259..43957); ORI23036.1
**NBRC 101979**	NZ_BJUH01000044.1 (c12147..6439); GEK40808.1			NZ_BJUH01000007.1 (88181..93601); WP_186808032.1	NZ_BJUH01000007.1 (80920..87639); WP_154962277.1
**FAM 20446**		NZ_VBSQ01000038.1 (5827..11709); WP_138426799.1			
**867_LCAS**		NZ_JUPZ01000131.1 (30281..36148); WP_049171665.1	NZ_JUPZ01000031.1 (3419..8002); WP_049169329.1		
**UW4**	NZ_AFYS01000067.1 (c77544..71836); WP_003603181.1			NZ_AFYS01000027.1 (c25696..20267), WP_003601280.1	NZ_AFYS01000027.1 (c19725..13285); WP_003601277.1
**A2-362**	NZ_AFYM01000071.1 (c7387..1679); WP_003580674.1			NZ_AZOE01000027.1 (20156..25780); WP_027111531.1	NZ_AFYM01000018.1 (c29384..22665); WP_003577862.1

**YNF-5**		SDJZ01000009.1 (60156..66038); RXS57989.1			
**BCRC 80156**		VBWM01000009.1 (10305..16187); TLF36687.1			
**BCRC 17487**		VBWL01000009.1 (10305..16187); TLF35258.1			
**UBLC-42**		JADPYW010000009.1 (10151..16033); MBI6597216.1			
**Z11**	MPOP01000032.1 (1418..7126); OJF73894.1			MPOP01000044.1; (491..5920); OJF73652.1	MPOP01000044.1 (6462..12977); OJF73653.1
**UW1**	NZ_JDWK01000063.1 (c12240..6532); WP_003585464.1			JDWK01000013.1 (18949..24378); NMN61755.1	NZ_JDWK01000013.1 (24920..31618); WP_003583360.1
**B900021**			NZ_LOJN01000127.1 (679..5253); WP_075761197.1		
**21/1**	AFYK01000040.1 (c11351..5643); EKQ00566.1				

1.3 Primer design

Primers were designed with Primer 3 and listed in Table S3.

Table S3. Primer sequences, their targets, and the annealing temperatures used for *L. casei prtP* and *prtR* gene screening and sequencing. Abbreviation: na, not applicable.

Gene	Primer	Sequence (5'->3')	Tm (℃)	Primer position	Description	Reference sequence (coordinates)	Expected length (bp)
PrtP	cas_PrtP1_F1	GGTGCTAACGGGACAGGTGA		868-888	specific for *prtP1* paralogous gene; used for PCR screening	NZ_CP006690.1 (2245339..2239631)	1,685
	cas_PrtP1_R1	CCACCAGCAGGACTATAAGTGAT		2530-2553			
	cas_PrtP2_F1	CCGATACAACAAGCGCCGTC		164-184	specific for *prtP2* paralogous gene; used for PCR screening and amplicon sequencing	NZ_CP017065.1 (2282143..2288025)	2,630
	cas_PrtP2_R1	CTGATTGGTCGAGCTGCTAAGC		2772-2794			
	cas_PrtPT_F	AAGCATGGCCGGTATTTTAC		739-759	common primers designed on conserved regions	NZ_CP017065.1 (82282143..2288025)	1,673
	cas_PrtPT_R	ACAAATTGCTGTTGGTCAAAAGA		2389-2412			
	cas_PrtP2_F2	CGGATGTCGAACAATTTACCA		na	specific for *prtP2* paralogous gene; used for as internal primer for PCR amplicon walking	NZ_CP017065.1 (2282143..2288025)	na
	cas_PrtP2_R2	CGAGACGATGACGTTGTTGTA					
PrtR	cas_PrtR1_F1	GGATTCCCAGATGGACGGCTTT		1326-1348	specific for *prtR1* paralogous gene; used for PCR screening and amplicon sequencing	NZ_CP017065.1 (2903816..2908414)	1,723
	cas_PrtR1_R1	GTCGGAAACCGCTTGAACGAAG		3027-3049			
	cas_PrtR1_F2	CGCAAACATATGCCGTCGAGTC		na			na

cas_PrtR1_R2	GTCTTGATAGCTTAGGCCGGTT		specific for *prtP2* paralogous gene; used for as internal primer for PCR amplicon walking	NZ_CP017065.1 (2903816..2908414)	
cas_PrtR2_F	ATCAGCGGCCAATCAAGTCGAAG	297-320	specific for *prtR2* paralogous gene; used for PCR screening	NZ_BJUH01000007.1 (88181..93601)	1,397
cas_PrtR2_R	TGATAGTAAACGTTGGTGCTGCCT	1670-1694			

2. Supplementary Results

2.1 Species confirmation

Closely related species such as *L. casei*, *L. paracasei*, and *L. rhamnosus* can be difficult to discriminate by conventional 16S rRNA gene-based barcoding methods. Strains PRA205 and 2006 were isolated from two different Parmigiano Reggiano cheeses during distinct isolation projects in 2011 and 2018, respectively. Strain PRA205 was attributed to *L. casei* species based on 16S-ARDRA [14], species-specific PCR [15] and 16S rRNA gene sequencing [4]. Strain 2006 was initially attributed to *L. rhamnosus* based on *tuf* gene-targeted PCR assay [16] and 16S rRNA gene sequencing. Before beginning Prt-encoding gene and protein characterization, we confirmed species attribution of the candidate strains by *mutL* gene targeted multiplex PCR. This method was recently proven to overcome *tuf* gene-targeted PCR and 16S rRNA gene-based barcoding methods to discriminate closely related species of the *L. casei* group [1]. Phylogenetic analysis of *mutL* nucleotide sequences available in GenBank showed that *L. casei* diverges in *mutL* sequences from *L. zeae* (Supplementary Figure S1). In 2020 *L. zeae* was restored as distinct species from *L. casei* [17]. Supplementary Figure S2 showed that, like PRA205, also strain 2006 belongs to *L. casei*.

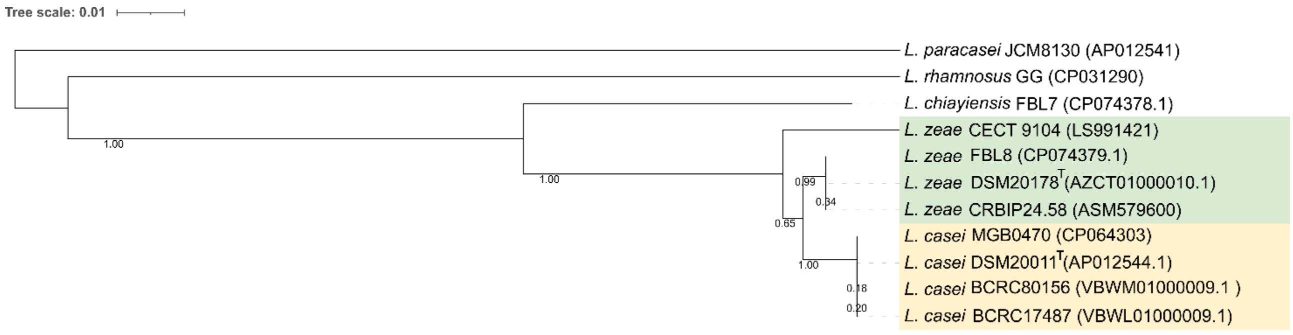

Figure S1. Phylogenetic relationships among *Lacticaseibacillus casei* species inferred by neighbor joining (NJ) method from *mutL* gene partial sequences. The GenBank accession numbers were reported in the brackets. The evolutionary distances were computed using the Kimura 2-parameter method and are in the units of the number of base substitutions per site. The rate variation among sites was modeled with a gamma distribution. Branch lengths are proportional to the numbers of nucleotide substitutions and are measured by the scale bar of sequence divergence. Bootstrap values are reported near to the branch.

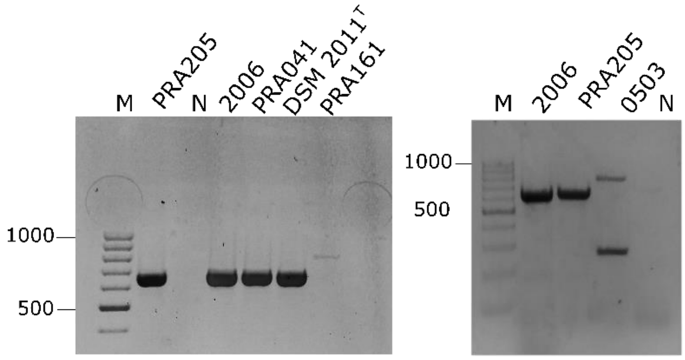

Figure S2. *mutL*-targeted PCR amplification of *L. casei* 2006 and PRA205. *Lacticaseibacillus casei* PRA041 and DSM 20011^{T} were used as positive controls, while *L. rhamnosus* PRA161 and 0503 as negative controls. Band sizes are in bp. Abbreviations: M, GeneRuler 100 bp DNA Ladders (Thermo Scientific, Waltham, USA); N, PCR mixture without any DNA templates.

Resequencing of 16S rRNA gene with internal primer WLAB2 allowed a proper coverage of V1-V2 regions and confirmed that strain 2006 does not belong to *L. rhamnosus* but formed a monophyletic group with *L. casei* ATCC 393^{T} (= DMS 20011^{T}) strictly related to *L. zeae* ATCC15820^{T} (Supplementary Figure S3).

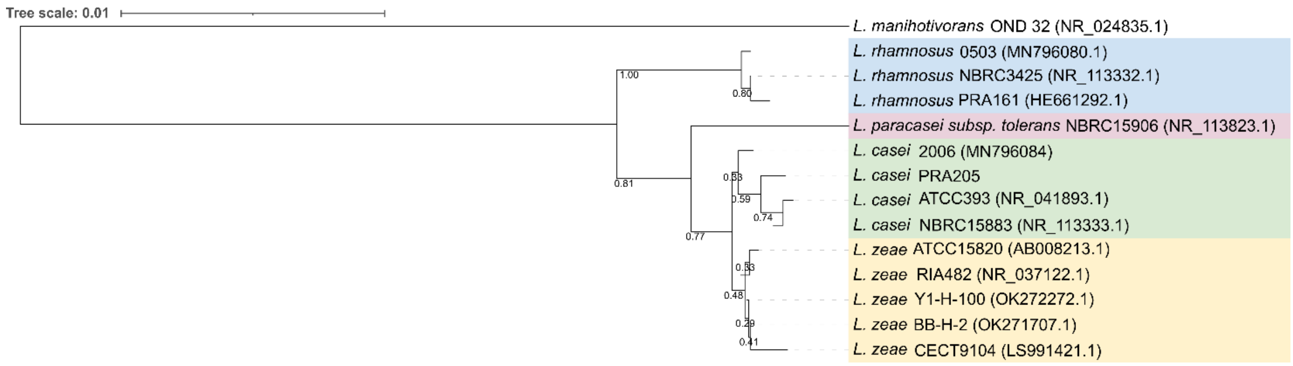

Figure S3. Phylogenetic tree generated from 1473 nucleotide positions of the strain 2006 16S rRNA gene. The tree was constructed using the Neighbor-joining method. The evolutionary distances were computed using the Kimura 2-parameter method and are in the units of the number of base substitutions per site. Bootstrap values (1000 replicates) of > 0.5 are indicated at the branching points, and GenBank accession numbers are shown in parentheses. The scale bar indicates a distance equivalent of one nucleotide change per 100 nucleotides.

2.2 Prt Domain identification and protein alignments

Figure S4 reported the summary of protein domains in PrtP1, PrtP2, PrtR1, PrtR2, and PrtR3 as revealed by Domain identification with Conserved Domains Database (CDD).

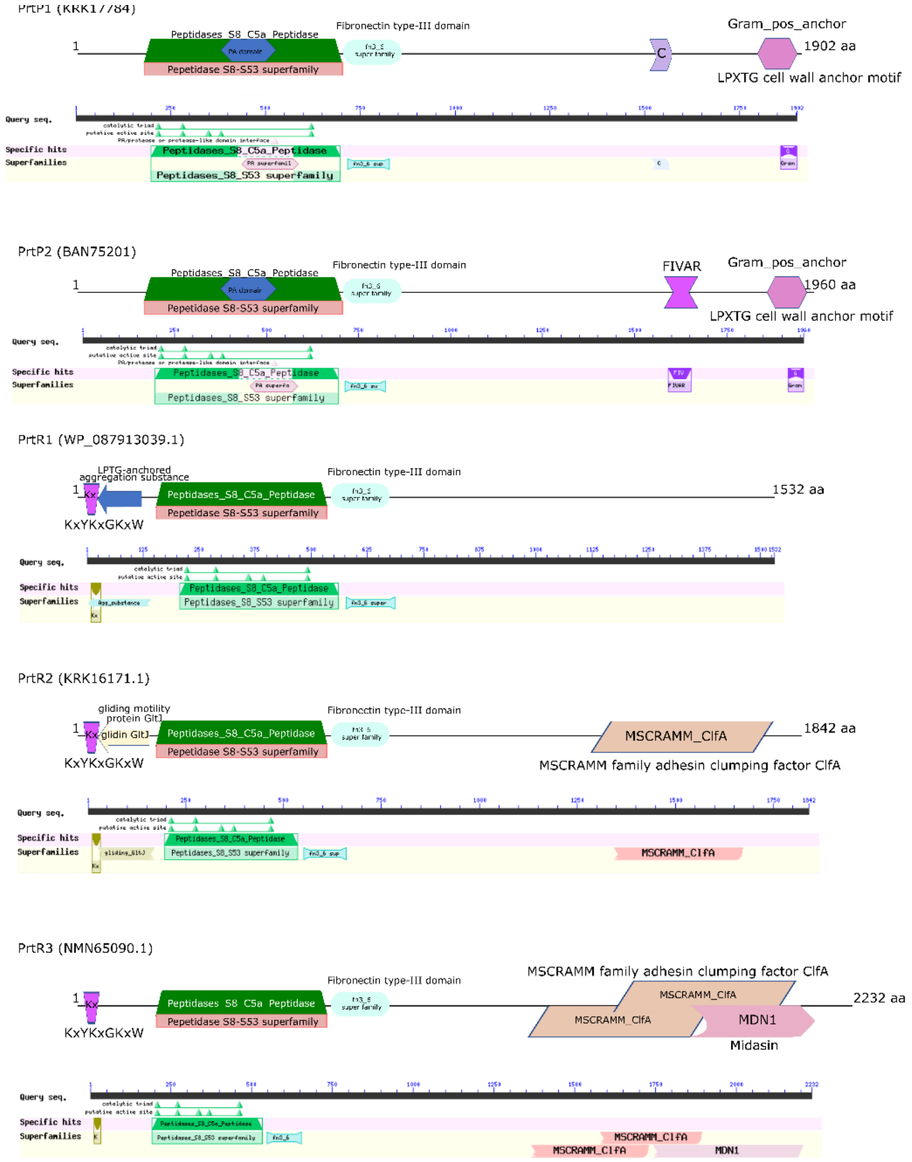

Figure S4. Representative selection of conserved domains identified in PrtP1, PrtP2, PrtR1, PrtR2, and PrtR3. Search for conserved domain was carried out with NCBI Batch Web Conserved Domain tool.

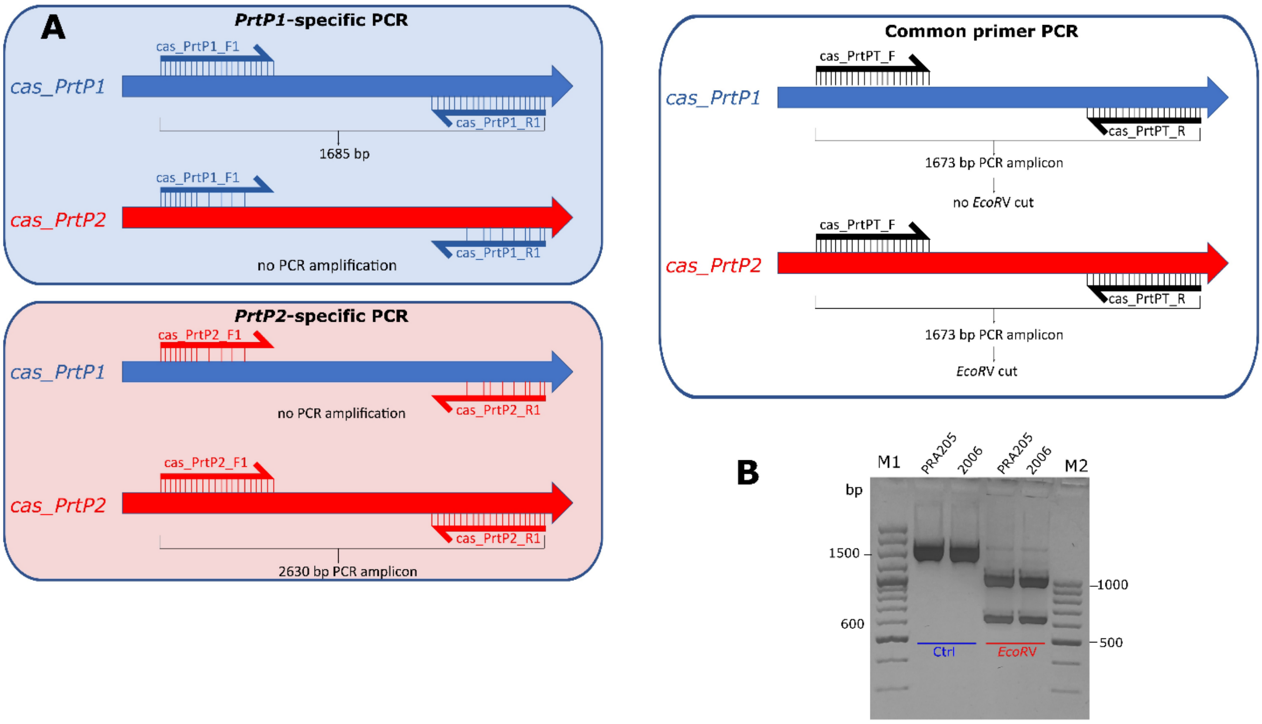

Figure S5. PCR-based approaches to dissect *PrtP* variants in *Lacticaseibacilluc casei* PRA205 and 2006. A. Schematic drawing of PCR strategies with *PrtP* variant-specific and common primers. Primer sequences were detailed in Table S3. B Restriction pattern of *prtP* gene PCR amplicons obtained with the conserved primers cas_PrtPT_F/cas_PrtPT_R. Primer pair was designed to amplify prtP1 and prtP2 genes, resulting in a PCR amplicon of app. 1,673 bp for both the target genes. Diagnostic endonuclease *EcoR*V was chosen to selectively cut *prtP2* paralogous gene resulting in two fragments of app. 1041 and 630 bp, respectively. M1 and M2 are GeneRuler 100 bp Plus DNA Ladders and GeneRuler 100 bp DNA Ladders (Thermo Scientific, Waltham, USA)

Polymorphic sites in Prt amino acid sequences of strain PRa205 and 2006 were reported in Figures S6 and S7.

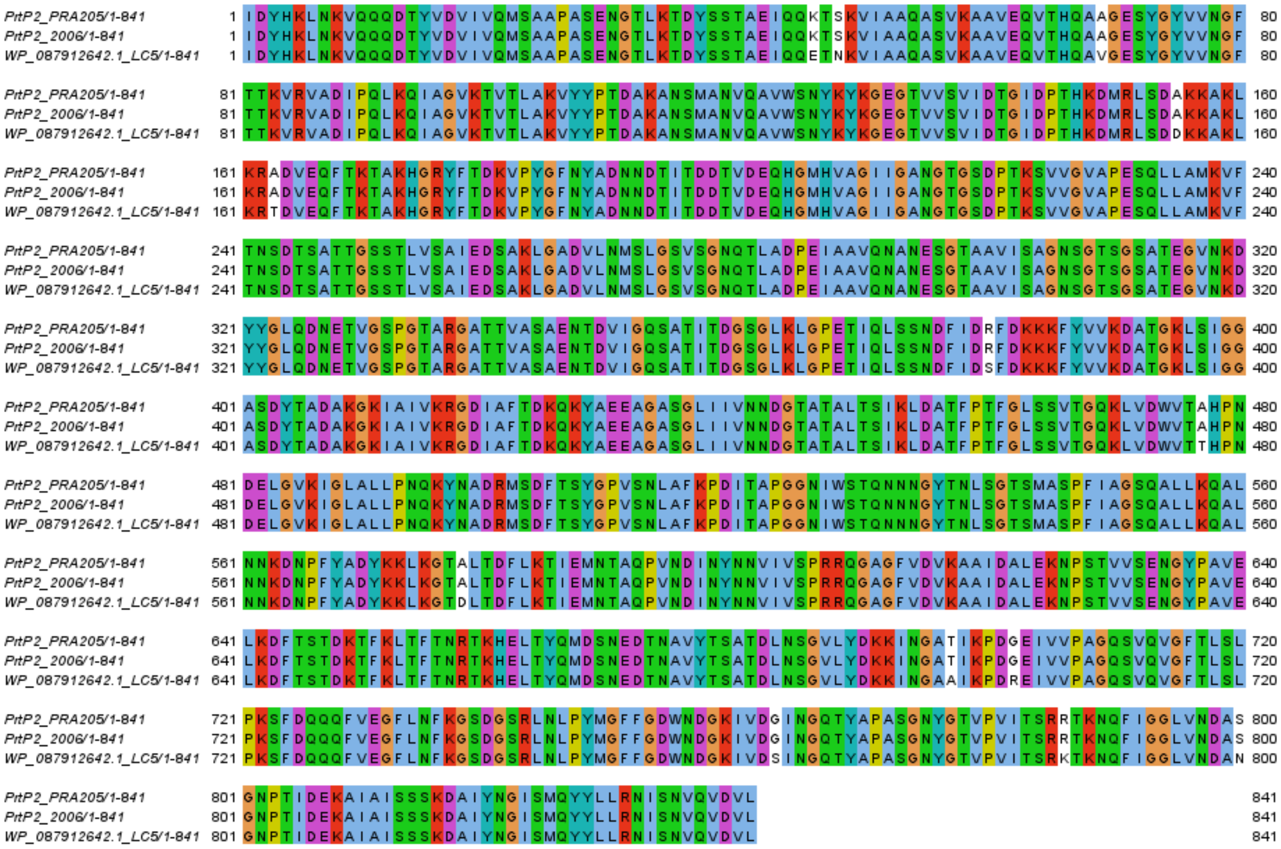

Figure S6. Multiple sequence alignment of PRA205 and 2006 PrtP2 partial proteins with PrtP2 reference protein from *Lacticaseibacillus casei* LC5. Alignment was don using Clustal Omega server and visualized using Jalview. The amino acid identities were coloured according to Clustal Omega color scheme.

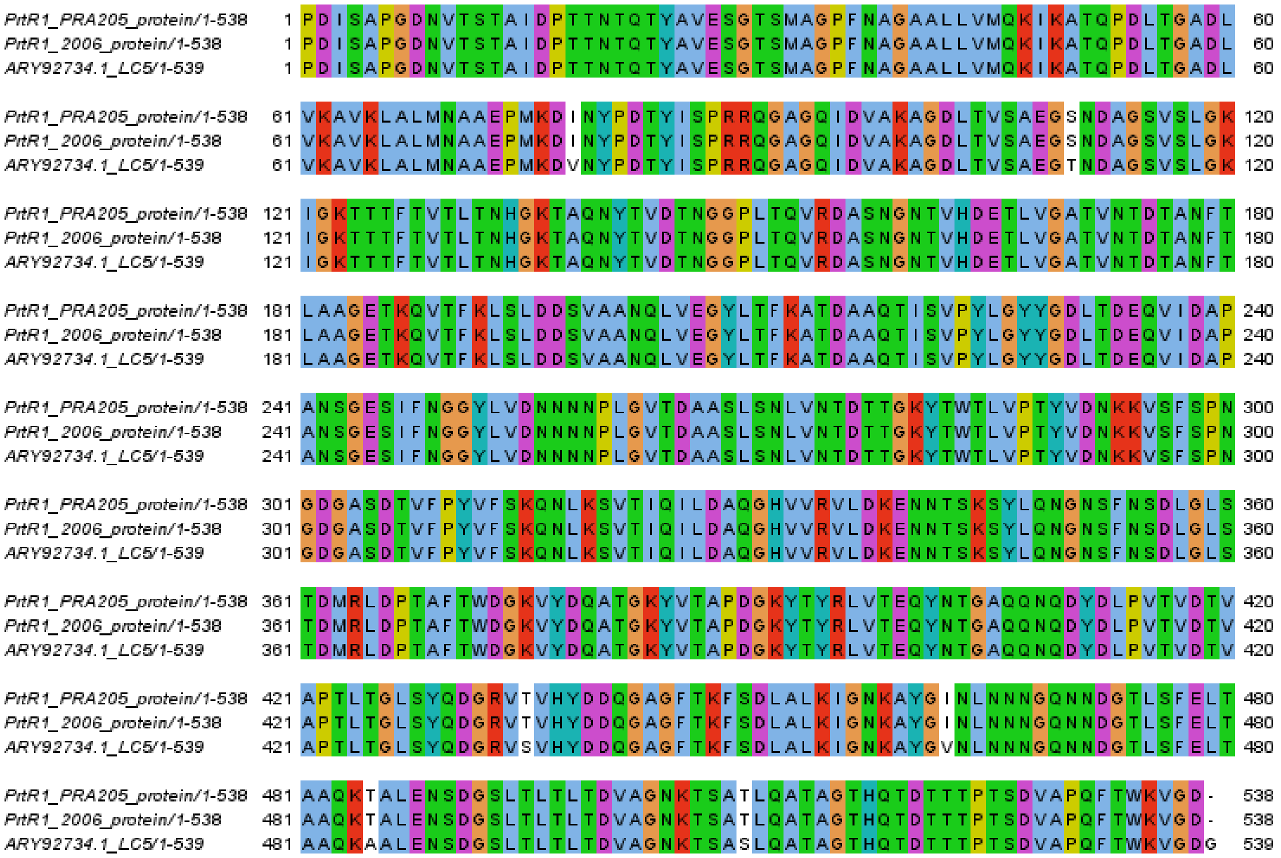

Figure S7. Multiple sequence alignment of PRA205 and 2006 PrtR1 partial proteins with Prtr1 reference protein from *Lacticaseibacillus casei* LC5. Alignment was don using Clustal Omega server and visualized using Jalview. The amino acid identities were coloured according to Clustal Omega color scheme.

2.3 Peptide releases

Number of peptides identified in β-casein and αS1-casein hydrolysates obtained PRA205 and 2006 PrtR1 proteinases were reported in Supplementary Figure S6, while differences between PRA205 and 2006 in peptide profiles were drawn as Venn diagrams (Supplementary Figure S7). Tables S3, S4, S5, and S6 listed all the peptides found in different hydrolysates by mass spectrometry.

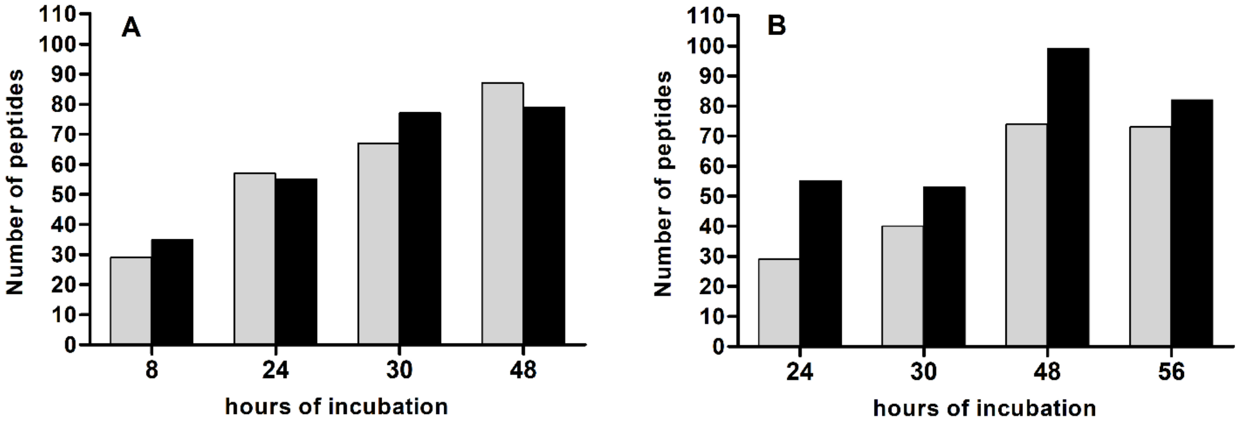

Figure S8. Number of peptides identified in β-casein and αS1-casein hydrolysates at the different time points. (A) Number of peptides identified in β-casein hydrolysates at 8, 24, 30 and 48 h of incubation. (B) Number of peptides identified in αS1-casein hydrolysates at 24, 30, 48 and 56 h of incubation. Grey columns identified the number of peptides released by *Lacticaseibacillus casei* PRA205 PrtR1 hydrolysis. Black columns identified the number of peptides released by *Lacticaseibacillus casei* 2006 PrtR1 hydrolysis.

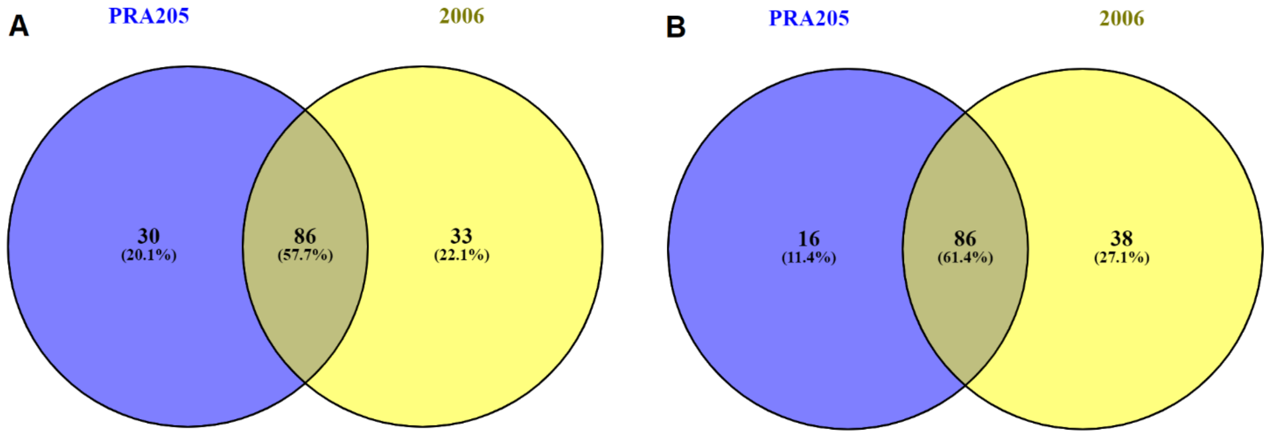

Figure S9. **Venn diagram displaying differences between peptides produced after β-casein (A) or aS1-casein (B) hydrolysis by *Lacticaseibacillus casei* PRA205 and 2006.** Venn diagrams were drawn using the online tool VENNY 2.1.0 by considering the peptides detected after 48 h and 56 h of β-casein and αS1-casein hydrolysis, respectively. See online Supplementary Material Table S4-S7 for the peptide sequences.

Table S4. List of peptides identified at the different time points during hydrolysis of β-casein with PrtR1 from *Lacticaseibacillus casei* PRA205

Fragment	*Sequence*	*Obseverd mass [M+H]*$^{+n}$	*Error (ppm)*	*8h*	*24h*	*30h*	*48h*
1 – 7	RELEELN	451.7334	1.93	-	+	+	+
1 – 31	RELEELNVPGEIVESLSSSEESITRINKKIE	962.6848	2.15	-	-	-	+
1 – 56	RELEELNVPGEIVESLSSSEESITRINKKIEKFQSEEQQQTEDELQDKIHPFAQTQ	1389.0224	0.29	-	-	-	+
32 – 46	KFQSEEQQQTEDELQ	973.9047	0.32	-	-	-	+
32 – 52	KFQSEEQQQTEDELQDKIHPF	895.4003	-0.45	-	-	-	+
32 – 56	KFQSEEQQQTEDELQDKIHPFAQTQ	1038.1321	-2.46	-	-	-	+
43 – 56	DELQDKIHPFAQTQ	835.4127	0.01	-	+	+	+
43 – 68	DELQDKIHPFAQTQSLVYPFPGPIPN	984.5037	0.96	-	-	-	+
43 – 72	DELQDKIHPFAQTQSLVYPFPGPIPNSLPQ	844.9363	0.64	-	-	+	-
47 – 57	DKIHPFAQTQS	636.3217	-1.45	-	-	-	+
47 – 68	DKIHPFAQTQSLVYPFPGPIPN	822.7647	-0.78	-	+	+	-
47 – 72	DKIHPFAQTQSLVYPFPGPIPNSLPQ	964.5112	3.46	-	+	+	+
57 – 68	SLVYPFPGPIPN	650.8511	0.99	-	-	-	+
65 – 67	PIP	326.2079	1.45	-	-	-	+
73 – 105	NIPPLTQTPVVVPPFLQPEVMGVSKVKEAMAPK	886.2464	3.14	+	-	-	-
73 – 99	NIPPLTQTPVVVPPFLQPEVMGVSKVK	729.4183	1.35	-	+	-	-
74 – 77	IPPL	439.2916	0.23	-	-	-	+
83 – 86	VVPP	411.2609	1.59	-	-	-	+
83 – 97	VVPPFLQPEVMGVSK	813.9525	3.28	-	-	-	+
92 – 97	VMGVSK	310.6759	1.38	-	+	+	+
94 – 105	GVSKVKEAMAPK	415.5733	1.79	-	+	-	-
97 – 105	KVKEAMAPK	334.5323	1.19	-	-	-	+
97 – 99	KVK	374.2765	0.77	-	-	+	+

100 – 128	EAMAPKHKEMPFPKYPVEPFTESQSLTLT	834.1695	0.88	-	-	-	+
100 – 132	EAMAPKHKEMPFPKYPVEPFTESQSLTLTDVEN	758.9746	2.59	-	-	-	+
100 – 139	EAMAPKHKEMPFPKYPVEPFTESQSLTLTDVENLHLPLPL	763.2293	1.02	-	+	-	-
103 – 105	APK	315.2029	0.74	-	-	-	+
103 – 124	APKHKEMPFPKYPVEPFTESQS	515.6606	1.55	-	-	-	+
103 – 128	APKHKEMPFPKYPVEPFTESQSLTLT	751.3892	0.55	-	-	-	+
103 – 139	APKHKEMPFPKYPVEPFTESQSLTLTDVENLHLPLPL	849.4486	-0.18	-	-	-	+
103 – 144	APKHKEMPFPKYPVEPFTESQSLTLTDVENLHLPLPLLQSWM	815.5909	-0.06	-	-	-	+
106 – 133	HKEMPFPKYPVEPFTESQSLTLTDVENL	819.9094	0.80	-	-	-	+
106 – 160	HKEMPFPKYPVEPFTESQSLTLTDVENLHLPLPLLQSWMHQPHQPLPPTVMFPPQ	1071.5456	-1.02	-	-	-	+
128 – 139	TDVENLHLPLPL	680.8792	2.91	-	+	+	+
129 – 139	DVENLHLPLPL	630.3530	-0.56	-	+	+	+
129 – 141	DVENLHLPLPLLQ	750.9230	-2.23	-	-	-	+
129 – 143	DVENLHLPLPLLQSW	887.4808	0.53	-	+	+	-
129 – 144	DVENLHLPLPLLQSWM	952.9975	-3.24	-	-	-	+
133 – 142	LHLPLPLLQS	565.8499	-0.62	-	-	-	+
134 – 142	HLPLPLLQS	509.3089	1.30	-	-	-	+
135 – 141	LPLPLLQ	397.2625	-0.56	+	+	+	+
135 – 142	LPLPLLQS	440.7797	2.25	+	+	+	+
144 – 156	MHQPHQPLPPTVM	756.8766	0.67	-	-	+	+
144 – 160	MHQPHQPLPPTVMFPPQ	661.3313	0.95	-	-	-	+
144 – 175	MHQPHQPLPPTVMFPPQSVLSLSQSKVLPVPQ	886.9745	-1.25	-	-	+	-
145 – 163	HQPHQPLPPTVMFPPQSVL	717.3814	3.72	-	-	-	+
151 – 154	LPPT	427.2559	1.77	-	+	+	+
161 – 175	SVLSLSQSKVLPVPQ	791.4668	3.31	+	+	+	+
161 – 176	SVLSLSQSKVLPVPQK	570.6795	4.63	+	+	+	-
161 – 182	SVLSLSQSKVLPVPQKAVPYPQ	789.1205	-0.91	+	+	-	-
161 – 183	SVLSLSQSKVLPVPQKAVPYPQR	631.1177	-0.44	-	+	+	-

161 – 209	SVLSLSQSKVLPVPQKAVPYPQRDMPIQAFLLYQEPVLGPVRGPFPIIV	1083.2121	-0.16	-	-	+	-
162 – 169	VLSLSQSK	431.2556	-0.12	-	-	+	-
162 – 175	VLSLSQSKVLPVPQ	747.9492	1.36	-	+	+	-
164 – 175	SLSQSKVLPVPQ	641.8738	3.02	+	+	+	+
164 – 176	SLSQSKVLPVPQK	705.9193	-0.06	-	+	+	+
164 – 182	SLSQSKVLPVPQKAVPYPQ	689.3938	1.14	+	+	+	+
164 – 183	SLSQSKVLPVPQKAVPYPQR	741.4272	0.62	-	+	+	+
164 – 186	SLSQSKVLPVPQKAVPYPQRDMP	642.1022	0.43	-	+	+	-
164 – 189	SLSQSKVLPVPQKAVPYPQRDMPIQA	959.8590	-1.07	-	-	-	+
164 – 190	SLSQSKVLPVPQKAVPYPQRDMPIQAF	1008.8814	-1.42	-	-	-	+
165 – 175	LSQSKVLPVPQ	598.3567	1.42	-	-	+	-
166 – 175	SQSKVLPVPQ	541.8160	3.90	+	+	+	+
166 – 176	SQSKVLPVPQK	404.2440	1.79	-	-	-	+
166 – 182	SQSKVLPVPQKAVPYPQ	622.6876	-0.11	-	+	+	+
166 – 183	SQSKVLPVPQKAVPYPQR	506.2944	3.07	-	+	+	+
167 – 183	QSKVLPVPQKAVPYPQR	645.7118	1.74	-	+	+	+
168 – 183	SKVLPVPQKAVPYPQR	452.5211	2.00	-	-	-	+
169 – 183	KVLPVPQKAVPYPQR	430.7622	0.12	-	-	-	+
170 – 182	VLPVPQKAVPYPQ	718.4202	1.69	-	+	+	+
170 – 183	VLPVPQKAVPYPQR	531.3147	-1.54	-	-	+	+
170 – 184	VLPVPQKAVPYPQRD	569.6589	2.01	+	+	+	+
170 – 186	VLPVPQKAVPYPQRDMP	645.6893	0.72	-	+	+	+
170 – 209	VLPVPQKAVPYPQRDMPIQAFLLYQEPVLGPVRGPFPIIV	1121.3834	-0.46	+	-	-	-
171 – 175	LPVPQ	553.3359	2.60	-	+	+	+
171 – 182	LPVPQKAVPYPQ	668.8831	-2.57	-	+	+	+
171 – 186	LPVPQKAVPYPQRDMP	612.6667	1.10	-	-	-	+
172 – 175	PVPQ	440.2500	-0.77	-	-	+	-
173 – 175	VPQ	343.1980	1.13	-	+	-	-

176 – 190	KAVPYPQRDMPIQAF	880.9605	-0.89	-	-	-	+
176 – 192	KAVPYPQRDMPIQAFLL	663.0324	-0.40	-	-	-	+
176 – 209	KAVPYPQRDMPIQAFLLYQEPVLGPVRGPFPIIV	1283.7138	-0.53	-	-	+	-
177 – 186	AVPYPQRDMP	587.2907	1.75	-	-	+	+
177 – 188	AVPYPQRDMPIQ	707.8593	-2.35	-	-	+	-
178 – 186	VPYPQRDMP	551.7717	1.07	-	-	+	+
178 – 190	VPYPQRDMPIQAF	781.3959	0.90	+	+	+	+
179 – 182	PYPQ	504.2455	0.37	-	-	-	+
180 – 188	YPQRDMPIQ	574.2842	4.10	-	+	+	+
180 – 190	YPQRDMPIQAF	683.3351	0.64	-	+	+	-
183 – 190	RDMPIQAF	489.2485	2.56	+	+	+	+
183 – 191	RDMPIQAFL	545.7910	3.14	-	+	+	-
183 – 192	RDMPIQAFLL	602.3327	2.33	-	+	+	-
183 – 193	RDMPIQAFLLY	683.8636	0.84	+	+	+	-
183 – 209	RDMPIQAFLLYQEPVLGPVRGPFPIIV	1022.5753	3.39	+	+	+	+
184 – 209	DMPIQAFLLYQEPVLGPVRGPFPIIV	1455.3094	4.02	-	-	-	+
186 – 188	PIQ	357.2137	1.32	+	+	+	-
190 – 206	FLLYQEPVLGPVRGPFP	965.0331	-2.22	+	-	-	-
191 – 193	LLY	408.2500	1.68	+	+	+	+
191 – 202	LLYQEPVLGPVR	692.4046	1.80	+	+	+	+
191 – 206	LLYQEPVLGPVRGPFP	891.5011	0.02	+	+	+	+
191 – 209	LLYQEPVLGPVRGPFPIIV	1054.1198	0.48	+	+	+	+
192 – 202	LYQEPVLGPVR	635.8622	1.33	-	-	-	+
192 – 204	LYQEPVLGPVRGP	712.8988	0.45	-	-	-	+
192 – 206	LYQEPVLGPVRGPFP	834.9612	2.53	+	+	+	+
192 – 209	LYQEPVLGPVRGPFPIIV	997.5768	-0.50	-	-	-	+
193 – 202	YQEPVLGPVR	386.5492	1.44	+	+	+	+
193 – 204	YQEPVLGPVRGP	656.3574	1.55	+	+	+	+

193 – 206	YQEPVLGPVRGPFP	778.4173	0.32	+	+	+	+
193 – 207	YQEPVLGPVRGPFPI	834.9621	3.65	+	+	+	+
193 – 209	YQEPVLGPVRGPFPIIV	941.0365	1.27	+	+	+	+
194 – 206	QEPVLGPVRGPFP	696.8869	2.29	+	+	+	+
194 – 209	QEPVLGPVRGPFPIIV	1717.9982	-1.03	-	-	+	+
195 – 206	EPVLGPVRGPFP	632.8556	-0.78	-	+	+	+
197 – 202	VLGPVR	320.7100	-2.18	-	+	+	-
199 – 206	GPVRGPFP	413.7328	1.55	-	-	+	-
207 – 209	IIV	344.2547	0.84	+	+	+	+

Table S5. List of peptides identified at the different time points during hydrolysis of aS1-casein with PrtR1 from *Lacticaseibacillus casei* PRA205

Fragment	*Sequence*	*Obseverd mass [M+H]+n*	*Error (ppm)*	*24h*	*30h*	*48h*	*56h*
1 – 8	RPKHPIKH	338.2097	-1.75	-	-	+	-
1 – 22	RPKHPIKHQGLPQEVLNENLLR	524.1039	2.35	+	+	+	+
1 – 23	RPKHPIKHQGLPQEVLNENLLRF	691.6447	1.46	+	+	+	+
1 – 24	RPKHPIKHQGLPQEVLNENLLRFF	582.9319	3.21	-	-	+	-
5 – 7	PIK	357.2497	0.31	+	+	+	+
8 – 16	HQGLPQEVL	510.7783	2.04	+	+	+	+
8 – 20	HQGLPQEVLNENL	745.8849	1.79	+	+	+	-
8 – 21	HQGLPQEVLNENLL	802.4264	1.02	-	+	+	+
8 – 22	HQGLPQEVLNENLLR	587.3198	0.03	+	+	+	+
8 – 23	HQGLPQEVLNENLLRF	636.3413	-2.06	+	+	+	+
10 – 22	GLPQEVLNENLLR	747.9183	1.27	+	+	+	+
10 – 23	GLPQEVLNENLLRF	547.9700	-0.27	-	+	+	+
10 – 24	GLPQEVLNENLLRFF	596.9960	5.07	-	-	+	+
11 – 22	LPQEVLNENLLR	719.4074	1.13	+	+	+	+
11 – 23	LPQEVLNENLLRF	528.9624	-1.16	-	+	+	+
11 – 24	LPQEVLNENLLRFF	866.4762	1.30	-	-	+	+
13 – 22	QEVLNENLLR	614.3369	-2.16	-	-	+	-
13 – 23	QEVLNENLLRF	687.8726	0.22	-	-	+	+
14 – 22	EVLNENLLR	550.3093	0.67	+	+	+	+
14 – 23	EVLNENLLRF	623.8438	1.07	+	+	+	+
14 – 24	EVLNENLLRFF	697.3770	-0.44	-	-	+	+
15 – 22	VLNENLLR	485.7883	1.47	+	+	+	+
15 – 23	VLNENLLRF	559.3222	0.56	+	+	+	+
16 – 18	LNE	375.1879	1.34	-	-	+	-

16 – 22	LNENLLR	436.2547	3.01	+	+	+	+
16 – 23	LNENLLRF	509.7885	1.82	+	+	+	+
17 – 22	NENLLR	379.7123	2.30	-	-	+	-
23 – 37	FFVAPFPEVFGKEKV	580.9852	2.85	-	-	-	+
24 – 35	FVAPFPEVFGKE	683.8563	0.77	-	+	+	+
24 – 42	FVAPFPEVFGKEKVNELSK	542.0482	3.18	-	-	+	-
25 – 34	VAPFPEVFGK	545.8016	2.47	-	-	+	-
25 – 35	VAPFPEVFGKE	610.3233	2.85	-	-	-	+
25 – 42	VAPFPEVFGKEKVNELSK	505.2801	1.46	-	-	-	+
26 – 35	APFPEVFGKE	560.7881	1.46	+	+	-	-
28 – 35	FPEVFGKE	476.7431	1.56	-	-	-	+
55 – 57	EDI	376.1722	1.95	-	+	-	-
80 – 82	HIQ	397.2196	0.63	-	-	+	-
83 – 102	KEDVPSERYLGYLEQLLRLK	817.1197	-0.08	-	-	-	+
83 – 91	KEDVPSERY	374.8527	1.00	-	-	-	+
83 – 93	KEDVPSERYLG	431.5550	1.90	-	+	+	+
85 – 100	DVPSERYLGYLEQLLR	651.0151	1.27	-	-	+	+
85 – 102	DVPSERYLGYLEQLLRLK	731.4075	0.28	-	-	-	+
87 – 102	PSERYLGYLEQLLRLK	660.0419	-0.48	-	-	-	+
91 – 100	YLGYLEQLLR	634.3567	1.36	-	-	-	+
91 – 102	YLGYLEQLLRLK	503.6329	0.39	-	-	-	+
92 – 100	LGYLEQLLR	552.8257	2.63	-	-	+	+
92 – 101	LGYLEQLLRL	609.3684	3.59	-	-	-	+
92 – 102	LGYLEQLLRLK	449.2780	-0.55	-	-	+	+
92 – 94	LGY	352.1874	2.03	-	+	-	-
93 – 100	GYLEQLLR	496.2829	1.48	-	-	+	+
93 – 102	GYLEQLLRLK	411.5838	0.60	-	-	+	+
94 – 100	YLEQLLR	467.7717	0.58	-	-	+	+

94 – 101	YLEQLLRL	524.3140	1.08	-	-	+	+
94 – 102	YLEQLLRLK	392.5771	1.79	-	+	+	+
95 – 100	LEQLLR	386.2398	0.18	-	-	+	+
95 – 101	LEQLLRL	442.7826	1.77	-	-	+	+
95 – 102	LEQLLRLK	506.8296	0.62	-	-	+	-
103 – 121	KYKVPQLEIVPNSAEERLH	777.4015	-0.24	-	-	-	+
103 – 123	KYKVPQLEIVPNSAEERLHSM	637.8217	0.85	-	-	+	+
103 – 128	KYKVPQLEIVPNSAEERLHSMKEGIH	623.3179	-1.36	-	-	-	+
103 – 130	KYKVPQLEIVPNSAEERLHSMKEGIHAQ	663.1396	2.55	-	+	+	+
104 – 121	YKVPQLEIVPNSAEERLH	551.2798	0.84	-	-	+	+
104 – 123	YKVPQLEIVPNSAEERLHSM	605.7988	2.31	-	-	+	+
104 – 130	YKVPQLEIVPNSAEERLHSMKEGIHAQ	796.6464	-0.56	-	-	+	+
105 – 121	KVPQLEIVPNSAEERLH	680.3490	0.24	-	-	+	+
105 – 123	KVPQLEIVPNSAEERLHSM	753.0398	0.17	-	-	-	+
106 – 108	VPQ	343.1979	0.97	-	-	+	-
106 – 121	VPQLEIVPNSAEERLH	637.6515	1.46	-	-	+	+
106 – 123	VPQLEIVPNSAEERLHSM	710.3426	1.63	-	-	+	+
106 – 130	VPQLEIVPNSAEERLHSMKEGIHAQ	723.8571	-0.21	-	-	+	+
108 – 121	QLEIVPNSAEERLH	572.2753	-2.61	-	-	-	+
109 – 121	LEIVPNSAEERLH	529.5927	3.88	+	+	+	+
109 – 123	LEIVPNSAEERLHSM	602.2831	2.75	+	+	+	+
110 – 121	EIVPNSAEERLH	491.8969	1.93	-	-	+	-
110 – 123	EIVPNSAEERLHSM	564.5876	1.43	-	-	+	+
111 – 121	IVPNSAEERLH	448.8823	1.31	+	-	+	-
111 – 123	IVPNSAEERLHSM	521.5738	2.31	+	-	-	-
112 – 121	VPNSAEERLH	411.1873	0.61	-	-	+	-
112 – 123	VPNSAEERLHSM	725.3143	1.52	+	+	+	+
115 – 123	SAEERLHSM	570.2322	1.77	-	-	+	-

115 – 130	SAEERLHSMKEGIHAQ	476.4702	3.23	-	-	+	-
124 – 143	KEGIHAQQKEPMIGVNQELA	555.7917	0.37	-	-	+	-
124 – 148	KEGIHAQQKEPMIGVNQELAYFYPE	730.6157	2.27	+	+	+	+
124 – 151	KEGIHAQQKEPMIGVNQELAYFYPELFR	667.9455	3.06	-	+	-	-
124 – 152	KEGIHAQQKEPMIGVNQELAYFYPELFRQ	693.5575	3.37	+	-	-	-
131 – 143	QKEPMIGVNQELA	486.2540	0.75	-	-	-	+
131 – 151	QKEPMIGVNQELAYFYPELFR	858.1047	3.50	-	-	-	+
131 – 152	QKEPMIGVNQELAYFYPELFRQ	900.7876	-0.32	-	-	-	+
136 – 152	IGVNQELAYFYPELFRQ	696.3591	1.44	-	-	-	+
142 – 144	LAY	366.2028	1.38	+	+	+	+
146 – 152	YPELFRQ	476.7487	1.56	+	+	+	+
147 – 149	PEL	358.1974	0.51	-	-	+	+
166 – 168	YVP	378.2027	0.84	-	-	+	-
170 – 194	GTQYTDAPSFSDIPNPIGSENSEKT	885.7394	-0.80	-	-	-	+
170 – 199	GTQYTDAPSFSDIPNPIGSENSEKTTMPLW	1095.1737	-1.07	-	+	+	+
173 – 194	YTDAPSFSDIPNPIGSENSEKT	790.3646	0.54	-	+	-	-
175 – 194	DAPSFSDIPNPIGSENSEKT	702.3269	-0.42	-	+	-	-
179 – 194	FSDIPNPIGSENSEKT	867.9116	-1.18	+	+	+	-
179 – 199	FSDIPNPIGSENSEKTTMPLW	1182.0637	-0.94	+	+	+	-
181 – 194	DIPNPIGSENSEKT	750.8633	1.11	+	+	+	+
181 – 199	DIPNPIGSENSEKTTMPLW	1065.0123	-2.12	+	+	+	-
197 – 199	PLW	415.2335	-1.22	-	+	+	+

Table S6. List of peptides identified at the different time points during hydrolysis of β-casein with PrtR1 from *Lacticaseibacillus casei* 2006

Fragment	*Sequence*	*Obseverd mass [M+H]+n*	*Error (ppm)*	*8h*	*24h*	*30h*	*48h*
1 – 7	RELEELN	451.7333	1.74	-	-	+	+
1 – 31	RELEELNVPGEIVESLSSSEESITRINKKIE	962.6842	1.48	-	-	-	+
1 – 46	RELEELNVPGEIVESLSSSEESITRINKKIEKFQSEEQQQTEDELQ	1444.6275	-0.77	-	-	+	-
1 – 52	RELEELNVPGEIVESLSSSEESITRINKKIEKFQSEEQQQTEDELQDKIHPF	1303.3819	0.26	-	-	+	-
1 – 56	RELEELNVPGEIVESLSSSEESITRINKKIEKFQSEEQQQTEDELQDKIHPFAQTQ	1157.6858	-0.38	-	-	-	+
29 – 52	KIEKFQSEEQQQTEDELQDKIHPF	764.3559	-2.37	-	-	+	-
32 – 46	KFQSEEQQQTEDELQ	973.9053	0.95	-	-	-	+
32 – 52	KFQSEEQQQTEDELQDKIHPF	895.4002	-0.57	-	-	-	+
32 – 56	KFQSEEQQQTEDELQDKIHPFAQTQ	1038.1332	-1.39	-	-	-	+
43 – 56	DELQDKIHPFAQTQ	835.4124	-0.24	-	-	-	+
43 – 68	DELQDKIHPFAQTQSLVYPFPGPIPN	984.5049	2.2	-	-	+	+
43 – 72	DELQDKIHPFAQTQSLVYPFPGPIPNSLPQ	844.9368	1.21	-	-	-	+
47 – 56	DKIHPFAQTQ	592.8065	-0.15	-	-	+	-
47 – 68	DKIHPFAQTQSLVYPFPGPIPN	822.7658	0.53	-	+	+	+
47 – 72	DKIHPFAQTQSLVYPFPGPIPNSLPQ	964.512	4.29	-	+	+	+
47 – 96	DKIHPFAQTQSLVYPFPGPIPNSLPQNIPPLTQTPVVVPPFLQPEVMGVS	1358.7254	-0.13	-	-	+	-
57 – 68	SLVYPFPGPIPN	650.8511	1.03	-	-	-	+
73 – 99	NIPPLTQTPVVVPPFLQPEVMGVSKVK	972.2206	-0.055	-	-	+	+
83 – 97	VVPPFLQPEVMGVSK	813.9505	0.8	-	-	-	+
83 – 99	VVPPFLQPEVMGVSKVK	618.6927	4.18	-	-	+	+
92 – 97	VMGVSK	310.6755	0.17	+	+	+	+
94 – 102	GVSKVKEAM	474.7633	1.15	-	-	+	+
94 – 105	GVSKVKEAMAPK	415.573	1.02	+	+	+	-
95 – 100	VSKVKE	345.2136	1.15	-	-	+	-

97 – 105	KVKEAMAPK	334.5324	1.49	-	+	+	+
97 – 99	KVK	374.2764	0.52	-	-	+	+
98 – 105	VKEAMAPK	437.2478	2.47	-	-	-	+
100 – 105	EAMAPK	323.6657	2.12	-	-	-	+
100 – 133	EAMAPKHKEMPFPKYPVEPFTESQSLTLTDVENL	781.5897	0.3	-	-	+	-
100 – 139	EAMAPKHKEMPFPKYPVEPFTESQSLTLTDVENLHLPLPL	763.2287	0.2	-	-	+	-
102 – 105	MAPK	446.2439	1.65	-	+	-	-
103 – 105	APK	315.2028	0.41	-	+	-	-
103 – 124	APKHKEMPFPKYPVEPFTESQS	515.6616	3.55	-	-	-	+
103 – 139	APKHKEMPFPKYPVEPFTESQSLTLTDVENLHLPLPL	849.4494	0.69	-	-	-	+
103 – 163	APKHKEMPFPKYPVEPFTESQSLTLTDVENLHLPLPLLQSWMHQPHQPLPPTVMFPPQSVL	1170.7745	-0.34	-	-	-	+
106 – 163	HKEMPFPKYPVEPFTESQSLTLTDVENLHLPLPLLQSWMHQPHQPLPPTVMFPPQSVL	1121.4099	-0.73	-	-	-	+
128 – 139	TDVENLHLPLPL	680.8787	2.2	-	-	+	+
129 – 139	DVENLHLPLPL	630.3539	0.96	-	+	+	+
129 – 141	DVENLHLPLPLLQ	750.9232	-1.97	-	-	-	+
129 – 143	DVENLHLPLPLLQSW	887.4804	0.12	-	-	+	+
133 – 142	LHLPLPLLQS	565.8505	0.4	-	+	+	+
135 – 141	LPLPLLQ	397.2622	-1.39	+	+	+	+
135 – 142	LPLPLLQS	440.7796	1.97	+	+	+	+
144 – 156	MHQPHQPLPPTVM	504.9207	1.72	-	-	+	+
144 – 160	MHQPHQPLPPTVMFPPQ	661.3321	2.07	-	+	-	-
144 – 175	MHQPHQPLPPTVMFPPQSVLSLSQSKVLPVPQ	886.9753	-0.37	-	+	-	-
145 – 156	HQPHQPLPPTVM	461.2405	1.81	-	-	-	+
145 – 163	HQPHQPLPPTVMFPPQSVL	717.3793	0.79	-	-	+	+
145 – 175	HQPHQPLPPTVMFPPQSVLSLSQSKVLPVPQ	854.2176	2.46	-	-	+	-
161 – 175	SVLSLSQSKVLPVPQ	791.4661	2.48	+	+	+	+
161 – 176	SVLSLSQSKVLPVPQK	570.6782	2.42	+	+	+	-
161 – 182	SVLSLSQSKVLPVPQKAVPYPQ	789.1221	1.13	+	+	-	-

161 – 183	SVLSLSQSKVLPVPQKAVPYPQR	631.1193	2.11	+	+	+	-
162 – 175	VLSLSQSKVLPVPQ	747.9488	0.93	+	-	-	-
164 – 174	SLSQSKVLPVP	577.8418	-1.36	-	-	+	-
164 – 175	SLSQSKVLPVPQ	641.8737	2.83	+	+	+	+
164 – 176	SLSQSKVLPVPQK	470.9491	0.84	-	+	+	+
164 – 182	SLSQSKVLPVPQKAVPYPQ	689.3935	0.72	+	+	+	+
164 – 183	SLSQSKVLPVPQKAVPYPQR	741.4275	1.13	+	+	+	+
164 – 186	SLSQSKVLPVPQKAVPYPQRDMP	642.101	-1.44	-	-	-	+
164 – 190	SLSQSKVLPVPQKAVPYPQRDMPIQAF	1008.8825	-0.33	-	-	+	+
164 – 192	SLSQSKVLPVPQKAVPYPQRDMPIQAFLL	1084.2691	-2.88	-	-	+	-
164 – 209	SLSQSKVLPVPQKAVPYPQRDMPIQAFLLYQEPVLGPVRGPFPIIV	1278.9673	-0.009	-	-	-	+
165 – 167	LSQ	347.1926	0.33	+	-	-	-
166 – 175	SQSKVLPVPQ	541.8158	3.53	+	+	+	+
166 – 176	SQSKVLPVPQK	404.2441	1.89	-	-	-	+
166 – 182	SQSKVLPVPQKAVPYPQ	622.6891	2.36	+	+	+	+
166 – 183	SQSKVLPVPQKAVPYPQR	506.2939	2.16	-	+	+	+
167 – 182	QSKVLPVPQKAVPYPQ	593.6773	0.54	-	+	-	-
167 – 183	QSKVLPVPQKAVPYPQR	484.5359	2.23	+	+	+	+
169 – 175	KVLPVPQ	390.7526	0.16	-	-	-	+
169 – 183	KVLPVPQKAVPYPQR	430.7627	1.3	-	-	+	+
170 – 175	VLPVPQ	652.4025	-0.5	-	-	+	-
170 – 176	VLPVPQK	390.7531	1.35	-	-	-	+
170 – 182	VLPVPQKAVPYPQ	718.4192	0.26	-	-	+	+
170 – 183	VLPVPQKAVPYPQR	531.3156	0.25	-	+	+	+
170 – 186	VLPVPQKAVPYPQRDMP	645.6899	1.62	+	+	+	+
170 – 209	VLPVPQKAVPYPQRDMPIQAFLLYQEPVLGPVRGPFPIIV	1121.384	0.047	+	-	-	-
171 – 175	LPVPQ	553.3354	1.74	-	+	+	-
171 – 182	LPVPQKAVPYPQ	668.8829	-2.78	-	+	+	+

171 – 183	LPVPQKAVPYPQR	498.293	0.67	-	-	+	+
172 – 175	PVPQ	440.2508	0.92	-	+	-	-
176 – 188	KAVPYPQRDMPIQ	514.9427	2.51	-	+	-	-
176 – 190	KAVPYPQRDMPIQAF	880.9597	-1.79	-	-	+	-
176 – 209	KAVPYPQRDMPIQAFLLYQEPVLGPVRGPFPIIV	1283.7151	0.48	+	-	-	-
177 – 186	AVPYPQRDMP	587.2904	1.15	-	-	+	-
177 – 189	AVPYPQRDMPIQA	743.3809	1.86	-	+	-	-
178 – 186	VPYPQRDMP	551.7727	2.87	-	+	+	-
178 – 190	VPYPQRDMPIQAF	781.3957	0.56	+	+	+	-
179 – 182	PYPQ	504.2459	1.21	+	+	+	+
180 – 190	YPQRDMPIQAF	683.3353	0.99	-	-	+	-
183 – 189	RDMPIQA	830.4175	-1.66	-	-	-	+
183 – 190	RDMPIQAF	489.248	1.47	+	+	+	+
183 – 191	RDMPIQAFL	545.7908	2.75	-	+	-	-
183 – 192	RDMPIQAFLL	602.3327	2.25	+	+	+	-
183 – 193	RDMPIQAFLLY	683.8633	0.43	+	+	+	+
183 – 209	RDMPIQAFLLYQEPVLGPVRGPFPIIV	1022.571	-0.81	+	+	+	+
186 – 188	PIQ	357.2136	1.13	-	-	+	-
189 – 209	AFLLYQEPVLGPVRGPFPIIV	1163.1726	0.39	+	-	-	-
190 – 209	FLLYQEPVLGPVRGPFPIIV	1127.655	1.28	-	-	-	+
191 – 193	LLY	408.2499	1.51	+	+	+	+
191 – 202	LLYQEPVLGPVR	692.4041	1.09	+	+	+	+
191 – 206	LLYQEPVLGPVRGPFP	891.4999	-1.36	+	+	+	+
191 – 209	LLYQEPVLGPVRGPFPIIV	1054.1213	1.83	-	+	+	+
192 – 202	LYQEPVLGPVR	635.8621	1.2	-	-	-	+
192 – 206	LYQEPVLGPVRGPFP	834.9604	1.61	+	+	+	+
192 – 209	LYQEPVLGPVRGPFPIIV	997.5772	-0.085	-	-	-	+
193 – 202	YQEPVLGPVR	579.3201	1.39	+	+	+	+

193 – 204	YQEPVLGPVRGP	656.3571	1.11	-	+	+	+
193 – 206	YQEPVLGPVRGPFP	778.417	-0.017	+	+	+	+
193 – 207	YQEPVLGPVRGPFPI	834.9619	3.39	-	+	+	+
193 – 209	YQEPVLGPVRGPFPIIV	941.0336	-1.82	+	+	+	+
194 – 206	QEPVLGPVRGPFP	696.8859	0.85	+	+	+	+
194 – 207	QEPVLGPVRGPFPI	753.4273	-0.16	-	-	+	-
194 – 209	QEPVLGPVRGPFPIIV	859.5034	-0.24	-	+	+	+
195 – 206	EPVLGPVRGPFP	632.8557	-0.59	-	-	+	+
196 – 198	PVL	328.2235	1.24	-	-	-	+
200 – 202	PVR	371.2403	0.61	-	+	+	-
207 – 209	IIV	344.2545	0.31	+	+	+	+

Table S7. List of peptides identified at the different time points during hydrolysis of aS1-casein with PrtR1 from *Lacticaseibacillus casei* 2006

Fragment	*Sequence*	*Obseverd mass [M+H]+n*	*Error (ppm)*	*24h*	*30h*	*48h*	*56h*
1 – 7	RPKHPIK	438.2818	-1.19	+	+	+	+
1 – 9	RPKHPIKHQ	570.8400	-1.82	-	-	+	-
1 – 13	RPKHPIKHQGLPQ	384.7294	2.70	-	-	+	-
1 – 14	RPKHPIKHQGLPQE	416.9899	1.98	-	-	+	-
1 – 16	RPKHPIKHQGLPQEVL	376.2237	1.38	-	-	+	-
1 – 22	RPKHPIKHQGLPQEVLNENLLR	654.8769	0.51	+	+	+	+
1 – 23	RPKHPIKHQGLPQEVLNENLLRF	691.6442	0.83	+	+	+	+
1 – 24	RPKHPIKHQGLPQEVLNENLLRFF	582.9320	3.28	+	+	+	+
1 – 36	RPKHPIKHQGLPQEVLNENLLRFFVAPFPEVFGKEK	707.3972	3.06	+	-	-	-
3 – 22	KHPIKHQGLPQEVLNENLLR	473.4732	2.62	+	+	+	+
3 – 23	KHPIKHQGLPQEVLNENLLRF	628.3555	0.39	-	-	+	+
4 – 22	HPIKHQGLPQEVLNENLLR	559.5644	0.05	-	-	+	+
5 – 7	PIK	357.2495	-0.25	-	+	+	+
8 – 16	HQGLPQEVL	510.7781	1.62	-	-	+	+
8 – 17	HQGLPQEVLN	567.7997	1.68	+	+	+	-
8 – 20	HQGLPQEVLNENL	745.8849	1.84	+	+	+	+
8 – 21	HQGLPQEVLNENLL	802.4246	-1.17	+	+	+	+
8 – 22	HQGLPQEVLNENLLR	587.3198	-0.10	+	+	+	+
8 – 23	HQGLPQEVLNENLLRF	636.3415	-1.79	+	+	+	+
9 – 22	QGLPQEVLNENLLR	811.9472	0.67	+	-	-	-
10 – 22	GLPQEVLNENLLR	498.9454	-3.94	+	+	+	+
10 – 23	GLPQEVLNENLLRF	821.4505	-1.24	+	+	+	+
10 – 24	GLPQEVLNENLLRFF	894.9883	2.81	-	+	+	+
11 – 21	LPQEVLNENLL	641.3578	2.74	-	-	-	+

11 – 22	LPQEVLNENLLR	719.4075	1.19	+	+	+	+
11 – 23	LPQEVLNENLLRF	528.9628	-0.43	+	+	+	+
11 – 24	LPQEVLNENLLRFF	577.9872	2.49	-	-	+	+
12 – 22	PQEVLNENLLR	662.8641	-0.74	-	-	+	+
13 – 22	QEVLNENLLR	614.3383	0.22	+	+	+	+
13 – 23	QEVLNENLLRF	687.8720	-0.62	-	-	-	+
14 – 22	EVLNENLLR	550.3095	1.04	+	+	+	+
14 – 23	EVLNENLLRF	623.8433	0.22	+	+	+	+
14 – 24	EVLNENLLRFF	697.3778	0.63	-	-	+	-
15 – 22	VLNENLLR	485.7880	0.78	+	+	+	+
15 – 23	VLNENLLRF	559.3226	1.40	+	+	+	+
16 – 22	LNENLLR	436.2548	3.21	+	+	+	+
16 – 23	LNENLLRF	509.7888	2.25	+	+	+	+
17 – 24	NENLLRFF	526.7795	-0.62	-	+	-	-
23 – 35	FFVAPFPEVFGKE	757.3921	2.90	-	-	-	+
24 – 34	FVAPFPEVFGK	619.3329	-2.45	+	+	+	-
24 – 35	FVAPFPEVFGKE	683.8562	0.76	+	-	+	+
24 – 37	FVAPFPEVFGKEKV	531.9626	3.63	-	-	+	-
24 – 42	FVAPFPEVFGKEKVNELSK	542.0481	3.07	-	-	+	+
24 – 50	FVAPFPEVFGKEKVNELSKDIGSESTE	1048.4792	-0.96	-	-	+	-
25 – 34	VAPFPEVFGK	545.8014	2.17	+	+	+	+
25 – 36	VAPFPEVFGKEK	449.9150	-0.11	-	+	+	+
25 – 42	VAPFPEVFGKEKVNELSK	505.2802	1.55	-	-	+	+
26 – 34	APFPEVFGK	496.2652	-1.58	-	-	-	+
26 – 35	APFPEVFGKE	560.7867	-1.07	-	-	-	+
26 – 36	APFPEVFGKEK	624.8351	0.46	+	+	+	-
28 – 35	FPEVFGKE	476.7433	1.90	+	-	-	+
55 – 57	EDI	376.1719	1.35	+	-	-	-

80 – 82	HIQ	397.2197	0.70	+	+	+	-
83 – 102	KEDVPSERYLGYLEQLLRLK	817.1195	-0.40	-	-	-	+
83 – 90	KEDVPSER	480.2440	1.61	-	-	-	+
83 – 92	KEDVPSERYL	412.5477	1.52	-	-	-	+
83 – 94	KEDVPSERYLGY	485.9097	2.19	-	-	-	+
85 – 100	DVPSERYLGYLEQLLR	651.0151	1.23	-	-	+	-
85 – 102	DVPSERYLGYLEQLLRLK	731.4083	1.37	-	-	-	+
92 – 94	LGY	352.1873	1.61	+	-	-	-
92 – 100	LGYLEQLLR	552.8256	2.59	+	+	+	+
92 – 101	LGYLEQLLRL	609.3680	2.84	-	-	+	-
92 – 102	LGYLEQLLRLK	449.2783	0.17	-	-	+	+
93 – 100	GYLEQLLR	496.2827	1.12	+	+	+	+
93 – 102	GYLEQLLRLK	411.5833	-0.51	-	-	+	+
94 – 100	YLEQLLR	467.7722	1.61	+	+	+	+
94 – 101	YLEQLLRL	524.3148	2.51	+	+	+	+
94 – 102	YLEQLLRLK	392.5769	1.26	+	+	+	+
95 – 100	LEQLLR	386.2398	0.09	-	-	+	+
95 – 101	LEQLLRL	442.7824	1.43	+	+	+	+
95 – 102	LEQLLRLK	338.2222	0.59	-	+	+	-
101 – 123	LKKYKVPQLEIVPNSAEERLHSM	558.6950	1.50	-	-	-	+
101 – 130	LKKYKVPQLEIVPNSAEERLHSMKEGIHAQ	711.3734	-0.47	-	-	+	-
103 – 121	KYKVPQLEIVPNSAEERLH	777.4013	-0.42	-	-	+	-
103 – 123	KYKVPQLEIVPNSAEERLHSM	637.8215	0.49	+	+	+	+
103 – 128	KYKVPQLEIVPNSAEERLHSMKEGIH	778.8954	-1.67	-	-	+	+
103 – 129	KYKVPQLEIVPNSAEERLHSMKEGIHA	637.5265	0.42	-	-	+	-
103 – 130	KYKVPQLEIVPNSAEERLHSMKEGIHAQ	663.1395	2.41	-	+	+	+
104 – 121	YKVPQLEIVPNSAEERLH	531.2873	-0.85	-	-	+	+
104 – 123	YKVPQLEIVPNSAEERLHSM	605.7988	2.22	-	+	+	+

104 – 130	YKVPQLEIVPNSAEERLHSMKEGIHAQ	796.6464	-0.61	-	-	+	+
105 – 121	KVPQLEIVPNSAEERLH	510.5145	2.06	+	+	+	+
105 – 123	KVPQLEIVPNSAEERLHSM	753.0390	-0.98	-	-	+	+
105 – 130	KVPQLEIVPNSAEERLHSMKEGIHAQ	604.9054	-1.50	-	-	+	+
106 – 108	VPQ	343.1979	0.86	+	-	-	-
106 – 121	VPQLEIVPNSAEERLH	637.6511	0.81	-	-	+	+
106 – 123	VPQLEIVPNSAEERLHSM	710.3424	1.43	-	-	+	+
106 – 130	VPQLEIVPNSAEERLHSMKEGIHAQ	964.8069	-0.42	-	-	-	+
109 – 114	LEIVPN	684.3929	0.30	-	+	+	-
109 – 119	LEIVPNSAEER	668.8115	1.03	-	-	+	-
109 – 121	LEIVPNSAEERLH	529.5925	3.52	+	+	+	+
109 – 123	LEIVPNSAEERLHSM	602.2835	3.42	+	+	+	+
110 – 121	EIVPNSAEERLH	491.8969	1.92	-	-	+	+
110 – 123	EIVPNSAEERLHSM	564.5869	0.13	-	-	+	+
111 – 121	IVPNSAEERLH	448.8820	0.51	-	-	+	-
111 – 123	IVPNSAEERLHSM	521.5737	2.08	+	-	-	-
112 – 121	VPNSAEERLH	411.1876	1.21	-	-	+	-
112 – 123	VPNSAEERLHSM	725.3140	1.02	-	-	+	+
115 – 123	SAEERLHSM	570.2317	0.95	+	+	+	+
115 – 130	SAEERLHSMKEGIHAQ	476.4702	3.36	-	-	+	-
122 – 148	SMKEGIHAQQKEPMIGVNQELAYFYPE	785.1339	2.19	-	-	+	-
124 – 143	KEGIHAQQKEPMIGVNQELA	555.7914	-0.09	-	-	+	+
124 – 148	KEGIHAQQKEPMIGVNQELAYFYPE	730.6159	2.52	+	+	+	+
124 – 151	KEGIHAQQKEPMIGVNQELAYFYPELFR	667.9455	3.15	+	+	+	+
124 – 152	KEGIHAQQKEPMIGVNQELAYFYPELFRQ	693.5584	4.70	+	+	-	-
131 – 154	QKEPMIGVNQELAYFYPELFRQFY	1004.1675	2.37	-	-	+	+
132 – 143	KEPMIGVNQELA	664.8478	0.38	-	-	+	-
132 – 144	KEPMIGVNQELAY	746.3786	-0.87	-	-	+	-

136 – 152	IGVNQELAYFYPELFRQ	696.3595	2.07	-	-	-	+
142 – 144	LAY	366.2029	1.51	+	+	+	+
144 – 151	YFYPELFR	567.7864	3.34	-	-	+	-
146 – 152	YPELFRQ	476.7485	1.00	+	-	-	-
147 – 149	PEL	358.1973	0.20	-	-	+	+
149 – 157	LFRQFYQLD	615.3198	0.87	-	-	+	-
170 – 194	GTQYTDAPSFSDIPNPIGSENSEKT	885.7392	-1.03	-	-	+	-
170 – 199	GTQYTDAPSFSDIPNPIGSENSEKTTMPLW	1095.1746	-0.18	-	-	+	+
174 – 194	TDAPSFSDIPNPIGSENSEKT	736.0089	-1.12	-	-	+	-
174 – 199	TDAPSFSDIPNPIGSENSEKTTMPLW	1417.6614	-1.19	-	-	+	-
175 – 194	DAPSFSDIPNPIGSENSEKT	1052.9871	-0.03	+	-	+	-
179 – 194	FSDIPNPIGSENSEKT	867.9111	-1.81	+	+	+	+
179 – 199	FSDIPNPIGSENSEKTTMPLW	1182.0642	-0.53	+	+	-	-
181 – 194	DIPNPIGSENSEKT	750.8635	1.43	+	+	+	+
181 – 199	DIPNPIGSENSEKTTMPLW	1065.0126	-1.87	+	+	+	+
197 – 199	PLW	415.2342	0.55	+	+	-	-

3. References

[1] Bottari, B., Felis, G.E., Salvetti, E., Castioni, A., Campedelli, I., Torriani, S., Gatti, M. (2017). Effective identification of *Lactobacillus casei* group species: genome-based selection of the gene mutL as the target of a novel multiplex PCR assay. *Microbiol* **2017**, 163, 950-960.

[2] Tagliazucchi, D., Baldaccini, A., Martini, S., Bianchi, A., Pizzamiglio, V., Solieri, L. Cultivable non-starter lactobacilli from ripened Parmigiano Reggiano cheeses with different salt content and their potential to release anti-hypertensive peptides. *Int J Food Microbiol* **2020**, *330*, 108688.

[3] Solieri, L., Bianchi, A., Giudici, P. Inventory of non-starter lactic acid bacteria from ripened Parmigiano Reggiano cheese as assessed by a culture dependent multiphasic approach. *Syst Appl Microbiol* 2012, **35**, 270–277.

[4] Sato, H., Yanagida, F., Shinohara, T., Yokotsuka, K. Restriction fragment length polymorphism analysis of 16S rRNA genes in lactic acid bacteria isolated from red wine. *J. Biosci. Bioeng.* **2000**, *90*, 335–337.

[5] Lopez, I., Ruiz-Larrea, F., Cocolin, L., Orr, E., Phister, T., Marshall, M., VanderGheynst, J., Mills, D.A. Design and evaluation of PCR primers for analysis of bacterial populations in wine by denaturing gradient gel electrophoresis. *Appl. Env. Microbiol.* **2003**, *69*, 6801-6807.

[6] Thompson, J.D., Higgins, D.G., Gibson, T.J. CLUSTAL W: improving the sensitivity of progressive multiple sequence alignment through sequence weighting, position-specific gap penalties and weight matrix choice. *Nucleic Acids Res.* **1994**, *22*, 4673–4680.

[7] Saitou N., Nei M. The neighbor-joining method: A new method for reconstructing phylogenetic trees. *Mol. Biol. Evol.* **1987**, *4*, 406-425.

[8] Kimura M. A simple method for estimating evolutionary rate of base substitutions through comparative studies of nucleotide sequences. *J. Mol. Evol.* **1980**, *16*, 111-120.

[9] Felsenstein J. Confidence limits on phylogenies: An approach using the bootstrap. *Evolution* **1985**, *39*, 783-791.

[10] Tamura, K., Stecher, G., Peterson, D., Filipski, A., Kumar, S. MEGA6: Molecular Evolutionary Genetics Analysis version 6.0. *Mol. Biol. Evol.* **2013**, *30*, 2725-2729.

[11] Letunic, I., Bork, P. Interactive Tree Of Life (iTOL) v4: recent updates and new developments. *Nucleic Acid Res* **2019**, *47*, W256-W259.

[12] Papadopoulos, J.S., Agarwala, R. COBALT: constraint-based alignment tool for multiple protein sequences. *Bioinformatics* **2007**, *23*, 1073-1079.

[13] Waterhouse, A.M., Procter, J.B., Martin, D.M.A., Clamp, M., Barton. G.J. Jalview Version 2-a multiple sequence alignment editor and analysis workbench. *Bioinformatics*, **2009**, *25*, 1189-1191.

[14] Rodas, A.M., Ferrer, S., Pardo, I. 16S-ARDRA, a tool for identification of lactic acid bacteria isolated from grape must and wine. *System. Appl. Microbiol.* **2003**, *26*, 412–422.

[15] Ward, L.H.J., Timmins, M.J. Differentiation of Lactobacillus casei, Lactobacillus paracasei and Lactobacillus rhamnosus by polymerase chain reaction. *Lett. Appl, Microbiol.* **1999**, *29*, 90–92.

[16] Ventura, M., Canchaya, C., Meylan, V., Klaenhammer, T.R., Zink, R. Analysis, characterization, and loci of the tuf genes in Lactobacillus and Bifidobacterium species and their direct application for species identification. *Appl. Env. Microbiol.* **2003**, *69*, 6908-6922.

[17] Liu, D.D., Gu, C.T. Proposal to reclassify *Lactobacillus zhaodongensis*, *Lactobacillus zeae*, *Lactobacillus argentoratensis* and *Lactobacillus buchneri* subsp. *silagei* as *Lacticaseibacillus zhaodongensis* comb. nov., *Lacticaseibacillus zeae* comb. nov., *Lactiplantibacillus argentoratensis* comb. nov. and *Lentilactobacillus buchneri* subsp. *silagei* comb. nov., respectively and *Apilactobacillus kosoi* as a later heterotypic synonym of *Apilactobacillus micheneri*. *Int J Syst Evol*, **2020**, *70*, 6414-6417.
